# Zebrafish models of skeletal dysplasia induced by cholesterol biosynthesis deficiency

**DOI:** 10.1242/dmm.042549

**Published:** 2020-06-24

**Authors:** Rebecca A. Anderson, Kevin T. Schwalbach, Stephanie R. Mui, Elizabeth E. LeClair, Jolanta M. Topczewska, Jacek Topczewski

**Affiliations:** 1 Department of Pediatrics, Northwestern University Feinberg School of Medicine, Chicago, IL 60611, USA; 2 Developmental Biology Program, Stanley Manne Children's Research Institute, Ann and Robert H. Lurie Children's Hospital of Chicago, Chicago, IL 60611, USA; 3 Department of Biochemistry and Molecular Biology, Medical University of Lublin, Lublin 20-093, Poland; 4 Department of Biological Sciences, DePaul University, Chicago, IL 60614, USA

**Keywords:** Cholesterol, Chondrodysplasia punctata, Lss, Msmo1, Skeletal dysplasia, Zebrafish

## Abstract

Human disorders of the post-squalene cholesterol biosynthesis pathway frequently result in skeletal abnormalities, yet our understanding of the mechanisms involved is limited. In a forward-genetic approach, we have found that a late-onset skeletal mutant, named *koliber^nu7^*, is the result of a *cis*-acting regulatory mutation leading to loss of *methylsterol monooxygenase 1* (*msmo1*) expression within pre-hypertrophic chondrocytes. Generated *msmo1^nu81^* knockdown mutation resulted in lethality at larval stage. We demonstrated that this is a result of both cholesterol deprivation and sterol intermediate accumulation by creating a mutation eliminating activity of Lanosterol synthase (Lss). Our results indicate that double *lss^nu60^;msmo1^nu81^* and single *lss^nu60^* mutants survive significantly longer than *msmo1^nu81^* homozygotes. Liver-specific restoration of either Msmo1 or Lss in corresponding mutant backgrounds suppresses larval lethality. Rescued mutants develop dramatic skeletal abnormalities, with a loss of Msmo1 activity resulting in a more-severe patterning defect of a near-complete loss of hypertrophic chondrocytes marked by *col10a1a* expression. Our analysis suggests that hypertrophic chondrocytes depend on endogenous cholesterol synthesis, and blocking C4 demethylation exacerbates the cholesterol deficiency phenotype. Our findings offer new insight into the genetic control of bone development and provide new zebrafish models for human disorders of the cholesterol biosynthesis pathway.

## Abstract (precis)

**Summary:** Characterization of three new zebrafish mutants highlights the role of cholesterol biosynthesis genes in skeletogenesis and establishes zebrafish models for disorders of the post-squalene cholesterol biosynthesis pathway, including chondrodysplasia punctata.

## INTRODUCTION

The cholesterol biosynthesis pathway is one of the most complex biochemical pathways and consists of over 30 enzymatic steps (Ačimovič et al., 2016; Sharpe and Brown, 2013). It can be divided into two major sections: the pre-squalene pathway, which is involved in isoprenoid synthesis and contains the rate-limiting enzyme of cholesterol biosynthesis, HMG-CoA reductase, and the post-squalene pathway, which is devoted to sterol synthesis (Ačimovič et al., 2016; Sharpe and Brown, 2013) (Fig. S1). At least ten human disorders result from mutations in post-squalene pathway genes (Herman and Kratz, 2012; Porter and Herman, 2011; Rossi et al., 2015). These disorders are characterized by intellectual disabilities, behavioral problems, heart and genital malformations, eye defects, skin conditions and skeletal deformities (Jira, 2013; Porter and Herman, 2011). Skeletogenesis defects vary from mild to severe, and are seen in endochondral bones, which develop through mineralization of cartilage, and intramembranous bones, which develop directly from condensed mesenchymal cells (Kronenberg, 2003; Ornitz and Marie, 2002).

In a subset of the post-squalene pathway disorders, chondrodysplasia punctata (CDP) is observed (Jurkiewicz et al., 2013). This rare skeletal phenotype is characterized by the observation of dot-like calcium deposits, or punctate, within cartilage on radiographs (Irving et al., 2008; Jurkiewicz et al., 2013; Lykissas, 2013). A result of ectopic calcification, the punctate is most often observed at the end of long bones and within cartilage around joints and the vertebral column (Jurkiewicz et al., 2013). Our understanding of how mutations in the post-squalene cholesterol biosynthesis pathway lead to abnormal skeletogenesis and CDP is limited.

Here, we show that the loss of *methylsterol monooxygenase 1* (*msmo1*) expression within pre-hypertrophic chondrocytes, owing to a *cis*-acting *koliber^nu7^* (*kol^nu7^*) regulatory mutation, results in defective chondrocyte differentiation, irregular bone formation and ectopic ossification within growth plates. We show that loss of Msmo1, and that of Lanosterol synthase (Lss), is lethal in zebrafish larvae. Restoration of hepatic Msmo1 or Lss activity is sufficient for post-larval survival of corresponding mutants. Transgenically rescued mutants develop strong skeletal defects similar to those seen in *kol^nu7^*. We show that Msmo1 and Lss activity is needed for proper chondrocyte differentiation, especially in the formation of hypertrophic chondrocytes. Our results suggest that the observed phenotypes are not a result of loss of Indian hedgehog (Ihh) signaling activity within growth plates. The *msmo1^nu81^* mutant phenotype is likely to be the combined result of cholesterol depletion and toxic intermediate accumulation as the *lss^nu60^* mutant, with blocked sterol synthesis, is epistatic to *msmo1* and has a less-severe phenotype.

## RESULTS

### The *kol^nu7^* mutation results in late-onset skeletal deformities

**Fig. 1.**
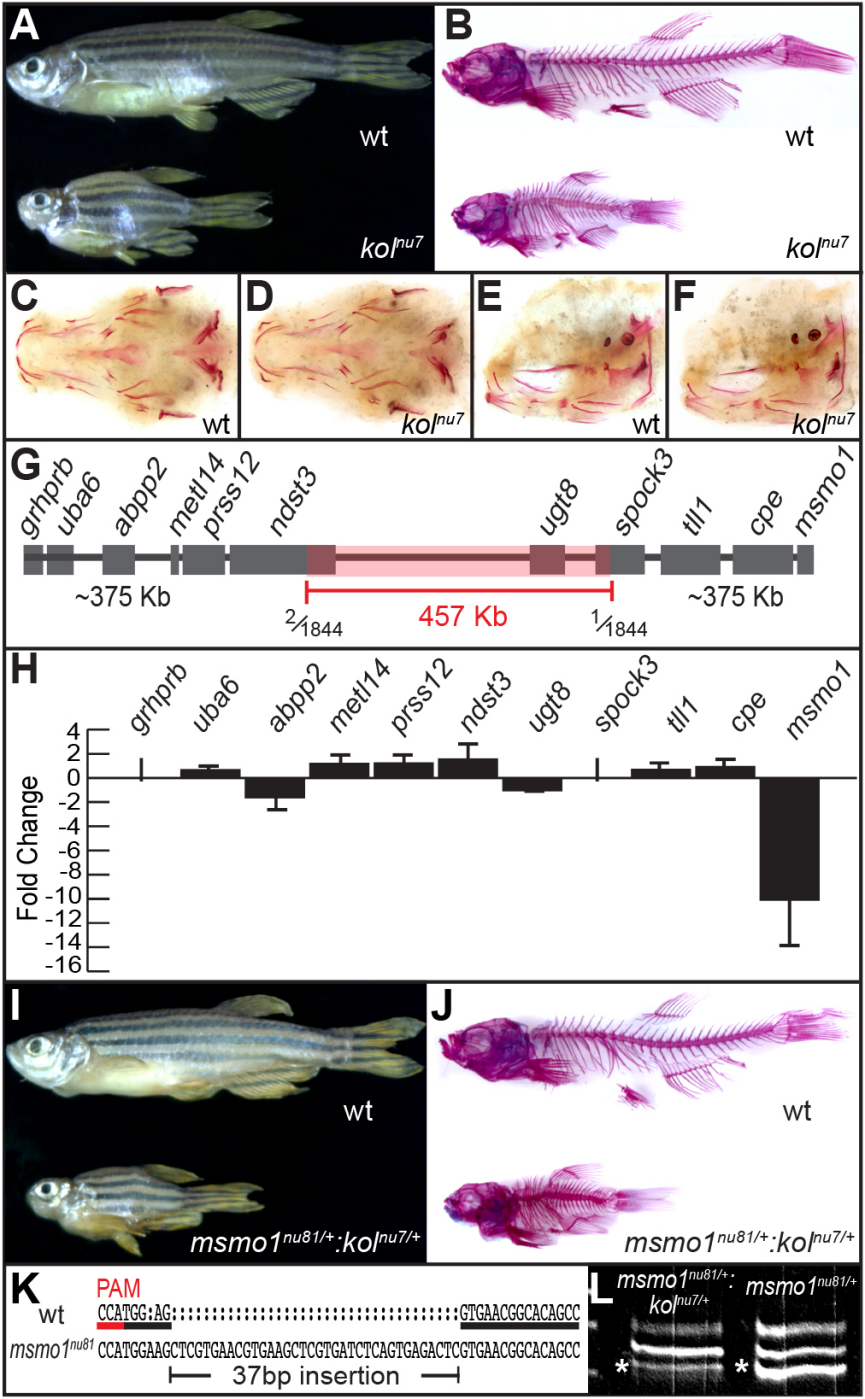
**Late-onset skeletal defects observed in the *koliber^nu7^* (*kol^nu7^*) mutant are the result of downregulation of *msmo1* expression.** (A) Compared to wild-type (wt) siblings (top), adult *kol^nu7^* mutants display a reduced body length and small head size. wt *n*=300, *kol^nu7^ n*=300. (B) Whole-mount skeletal preparations reveal gross malformations and hyperossification throughout the adult *kol^nu7^* craniofacial and axial skeleton after Alcian Blue (cartilage) and Alizarin Red (ossified bone and mineralized tissues) staining. wt *n*=100, *kol^nu7^ n*=100. (C-F) Early larval mutants do not display patterning defects or premature ossification. Whole-mount Alizarin Red staining of 4.7 mm (∼8 dpf) wt (C,E) and *kol^nu7^*. (D,F). Ventral view (C,D) and lateral view (E,F). wt *n*=3, *kol^nu7^ n*=4. (G) Positional cloning reveals that the *kol^nu7^* locus is located to the ∼457 kb critical region flanked by polymorphic markers with one or two recombinants out of 1844 meioses, corresponding to a genetic distance of 0.16 cM. (H) Screen of gene expression using quantitative RT-PCR from RNA extracted from hypural complex of ∼18 mm SL *kol^nu7^* and wt siblings. wt *n*=2, *kol^nu7^* =2. Only *msmo1* level was significantly different out of 11 tested genes, located in the ∼1.2 Mb region encompassing the *kol^nu7^* locus. Initial screen results: *grhprb* not detected (ND); *uba6* mean difference 0.64, s.d. 0.36; *abpp2* −1.62, s.d. 1.00; *mettl14* 1.16, s.d. 0.68; *prss12* 1.20, s.d. 0.72; *ndst3* 1.51, s.d. 1.50; *ugt8* −1.03, s.d. 0.07; *spock3* ND; *tll1* 0.67, s.d. 0.27; *cpe* 0.90, s.d. 0.42; *msmo1* −10.07, s.d. 4.32. Confirmation test of *msmo1* expression (*msmo1* −11.47, s.d. 8.49; *P*=0.0012), wt *n*=3, *kol^nu7^* =5. Three technical replicates were included for all assays. Gene expression was normalized to the reference gene *eefla1*. Fold change was calculated using Livak method (Livak and Schmittgen, 2001). *P*-value calculated using unpaired Student's *t*-test on dCt values. (I) The *msmo1^nu81^* mutant allele is not able to complement the *kol^nu7^* mutation. Adult *kol^nu7/+^:msmol^nu81/+^* transheterozygotes phenocopy the *kol^nu7^* mutant. wt *n*=100, *kol^nu7/+^:msmol^nu81/+^ n*=100. (J) Whole-mount skeletal preparations reveal gross malformations throughout the *kol^nu7/+^:msmol^nu81/+^* craniofacial and axial skeleton, similar to those observed in *kol^nu7^*. wt *n*=10, *kol^nu7/+^:msmo1^nu81/+^ n*=10. (K) The *msmo1^nu81^* allele is the result of a 37 bp insertion, allowing for allele-specific expression analysis between *msmo1^nu81^* and *kol^nu7^*. PAM, protospacer adjacent motif (underlined in red). (L) Strong downregulation of the *kol^nu7^*-linked allele (asterisks) in *kol^nu7/+^:msmo1^nu81/+^* compared to the wt allele in *msmo1^nu81/+^* suggests that the *kol^nu7^* mutation is *cis*-acting. *kol^nu7/+^:msmo1^nu81/+^ n*=3, *msmo1^nu81/+^ n*=3. The top band is a heterodimer of wt and mutant strands.

We identified a novel mutation, referred to as *kol^nu7^*, based on the reduced body length and small head size of adult homozygote fish (Fig. 1A). The *kol^nu7^* phenotype is first detectable by gross morphological examination at ∼6 weeks of development and the mutation is fully recessive. The *kol^nu7^* homozygote mutants are viable and fertile. Bone and cartilage staining of adult *kol^nu7^* using Alizarin Red and Alcian Blue, respectively (Fig. 1B), reveals defects in both endochondral and intramembranous bones (Bird and Mabee, 2003; Cubbage and Mabee, 1996; Parichy et al., 2009). Specifically, the endochondral bones of adult *kol^nu7^* mutants have dramatically reduced or missing growth plates. Interestingly, only the intramembranous bones located next to cartilaginous elements appear affected. For example, the dentary bone, which develops around Meckel's cartilage (Cubbage and Mabee, 1996), is significantly shortened in mutants relative to wild-type siblings (Fig. S2B,D) and the cartilage itself contains ectopic ossifications (Fig. S2D,D′). Similarly, the observable compressed body phenotype of *kol^nu7^* (Fig. 1A,B) is a result of partial or complete vertebral fusions (Fig. 1B; Fig. S3D,D′). In zebrafish, the vertebrae develop through direct mineralization around a relatively large cartilaginous notochord (Pogoda et al., 2018). In contrast, isolated intramembranous bones, such as the fin rays and operculum, appear relatively unaffected in *kol^nu7^* (Fig. 1B; Fig. S2C). Consistent with the late-onset phenotype of *kol^nu7^*, initial cartilage formation and patterning is normal (Fig. S4B), ossification is not prematurely initiated (Fig. 1C-F) and the initial patterning of vertebra centra ossification is normal (Fig. S3A,B).

Using positional cloning, we mapped the *kol^nu7^* mutation to a critical region located on chromosome 1 flanked by markers segregating in either one or two out of 1844 meioses (Fig. 1G). The critical region physical distance is ∼457 kb, while the genetic distance corresponds to 0.16 cM. As the average physical distance corresponds to ∼650 Mb per 1 cM (Talbot and Schier, 1999), this result indicates a greater than four times reduction in recombination frequency in this region. The critical region overlaps with three known genes (Fig. 1G). Coding sequence analysis of genes within the critical region, as well as those genes neighboring the critical region, did not reveal any changes in protein-coding sequence between wild-type and *kol^nu7^* siblings (data not shown). Using CRISPR/Cas9 genome editing, we mutagenized all three known genes within the critical region and found that each fully complemented the *kol^nu7^* mutation (Table 1; Fig. S5). These results suggested that the *kol^nu7^* mutation disrupts a regulatory sequence located within the critical region.

**Table 1.**
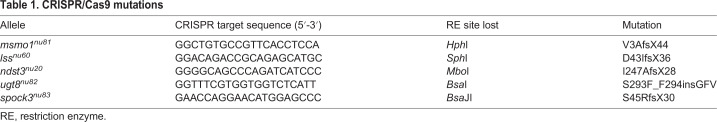
**CRISPR/Cas9 mutations**

### The *kol^nu7^* mutation negatively regulates expression of *msmo1*, a gene involved in cholesterol biosynthesis

To test the prediction that the *kol^nu7^* mutation disrupts a *cis*-acting regulatory element important for bone development, we assessed expression of the genes located in a ∼1.2Mb region containing the *kol^nu7^* locus (Fig. 1G,H). For quantitative real-time PCR (RT-PCR) analysis, we extracted total RNA from the hypural complexes of wild-type and *kol^nu7^* mutant fish. The hypural complex, composed of endochondral bones, is severely affected in *kol^nu7^* mutants. For our analysis, we selected fish that were ∼3 months old, corresponding to a standard length (SL) of ∼18 mm in wild-type siblings. Of the 11 genes analyzed, expression of *methylsterol monooxygenase 1* (*msmo1*) was more than 10-fold downregulated (Fig. 1H). Similarly, using RNA isolated from the cranial vault, which consists of both intramembranous and endochondral bones (Topczewska et al., 2016), we found a 3.8-fold downregulation of *msmo1* in *kol^nu7^*. Finally, a 2-fold downregulation of *msmo1* expression was detected in total RNA isolated from eviscerated trunks of *kol^nu7^* mutants. These results indicate that the *kol^nu7^* mutation induces a downregulation of *msmo1* expression, particularly in bone-enriched tissues.

**Fig. 2.**
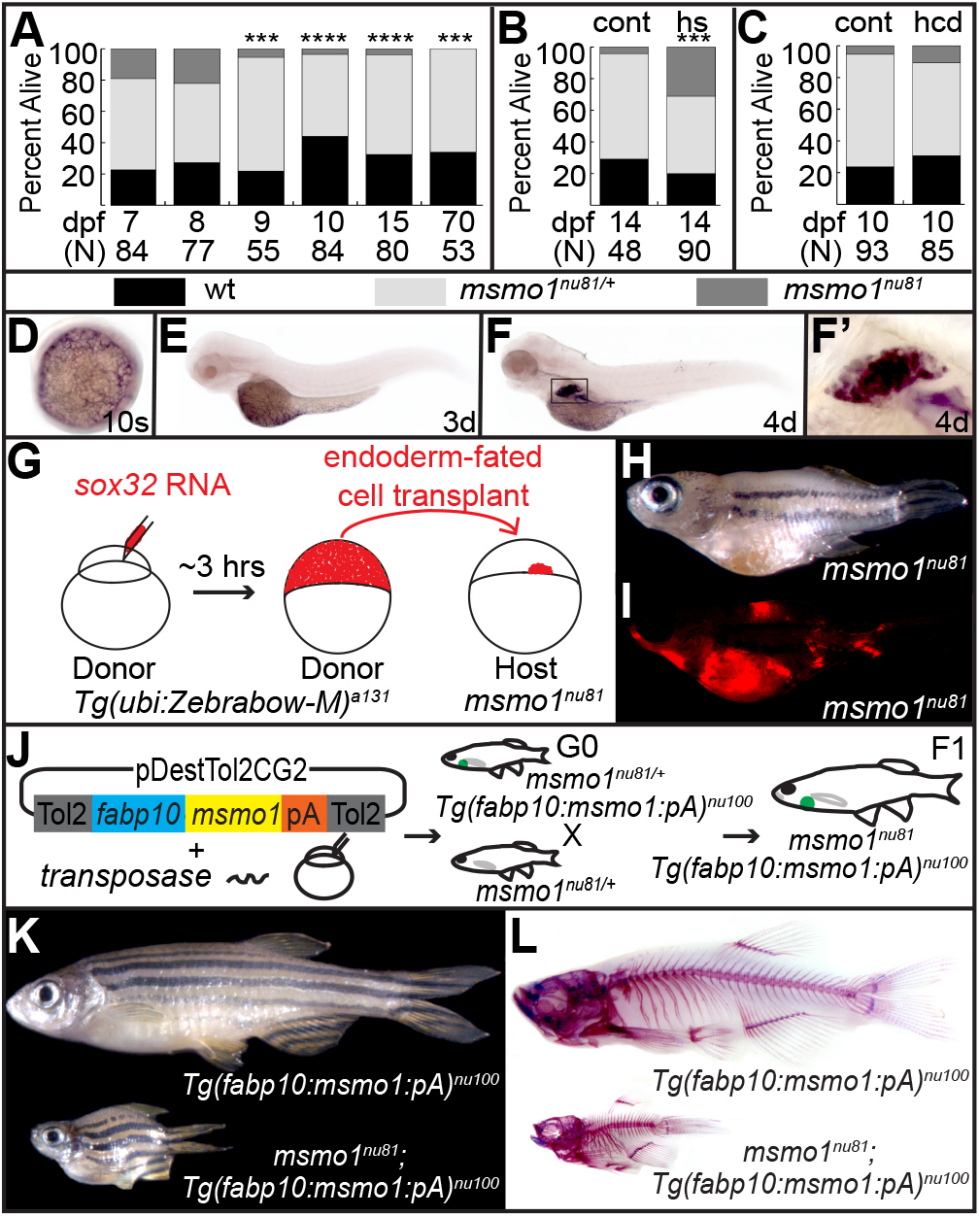
**The suppression of early lethality of the *msmo1^nu81^* mutation yields adult fish with *kol^nu7^*-like phenotype.** (A) Survival of larvae from *msmo1^nu81/+^* in-crosses, from 7 dpf to 70 dpf. Most *msmo1^nu81^* mutants die by 9 dpf. [7 dpf wild type (wt; black) *n*=19, heterozygotes (het; pale gray) *n*=49, knockout mutants (KO; dark gray) *n*=16, *P*=0.02794; 8 dpf wt *n*=21, het *n*=39, KO *n*=17, *P*=0.8065; 9 dpf wt *n*=12, het *n*=40, KO *n*=3, ***P=0.0008; 10 dpf wt *n*=37, het *n*=44, KO *n*=3, ****P<0.0001; 15 dpf wt *n*=26, het *n*=51, KO *n*=3, ****P<0.0001; 70 dpf wt *n*=18, het *n*=35, KO *n*=0, ***P=0.001.] (B) Overexpression of *msmo1* driven by daily heat shock of the transgenic line *Tg(hsp70l:msmo1:IRESnlsGFP)^nu99^* rescued the lethality of *msmo1^nu8^* mutants. Transgenic screening based on cardiac GFP. (Control non-transgenic siblings 14 dpf wt *n*=14, het *n*=32, KO *n*=2, ***P=0.0035; transgenic siblings 14 dpf wt *n*=18, het *n*=44, KO *n*=28, *P*=0.3214.) cont, control; hs, heat shock. (C) Dietary cholesterol supplementation does not improve the survivability of *msmo1^nu81^* mutants. Clutches from *msmo1^nu81/+^* in-crosses were fed either a high-cholesterol diet (hcd) or a control standardized diet (cont) beginning at 5 dpf until collection at 10 dpf. (HCD wt *n*=26, het *n*=50, KO *n*=9, *P*=0.0089; control diet wt *n*=22, het *n*=66, KO *n*=5, *P*<0.0001.) All two-tailed *P*-values were calculated using chi-squared test. (D-F′) Whole-mount *in situ* hybridization during the first 5 days of development shows *msmo1* expression predominately in the yolk syncytial layer (YSL) and liver. Expression is first detected during early somitogenesis in the YSL (D) and continues there at 3 dpf (E). At 4 dpf, strong expression is observed in the differentiated liver (F,F′). (G-I) Generation of *msmo1^nu81^*/wild-type chimeras using endoderm replacement rescues early lethality of *msmo1^nu81^* mutants and reveals a strong *kol^nu7^*-like phenotype. (G) Schematic of the procedure. Wild-type *Tg(ubi:Zebrabow-M)^a131^* donor embryos were injected with *sox32* RNA to force an endodermal fate. At high stage (∼3 hpf), cells were transplanted from donor to host embryos collected from *msmo1^nu81/+^* in-crosses. (H) Surviving *msmo1^nu81^* mutants display a strong *kol^nu7^*-like phenotype. *msmo1^nu81^ n*=4. (I) The majority of organs of endodermal origin displayed a high enrichment in transplanted *Tg(ubi:Zebrabow-M)^a131^* cells (red). *msmo1^nu81^ n*=4. (J-L) Liver-specific *msmo1* expression in *msmo1^nu81^* mutants rescues early lethality and produces juvenile *msmo1^nu81^* mutants with strong *kol^nu7^*-like phenotype. (J) Liver-specific regulatory element *fabp10a* was used to drive *msmo1* expression in *msmo1^nu81^* mutants. (K) Adult *Tg(fabp10a:msmo1:pA)^nu100^;msmo1^nu81^* mutants phenocopy *kol^nu7^* mutant. *Tg(fabp10:msmo1:pA)^nu100^ n*=50, *Tg(fabp10a:msmo1:pA)^nu100^;msmo1^nu81^ n*=50. (L) Whole-mount skeletal preparations reveal gross malformations throughout *Tg(fabp10a:msmo1:pA)^nu100^;msmo1^nu81^* craniofacial and axial skeleton, similar to those observed in *kol^nu7^*. *Tg(fabp10:msmo1:pA)^nu100^ n*=3, *Tg(fabp10a:msmo1:pA)^nu100^;msmo1^nu81^ n*=7.

The Msmo1 enzyme catalyzes the removal of a methyl group from C4-methlysterols during the post-squalene cholesterol biosynthesis pathway (Sharpe and Brown, 2013) (Fig. S1). To characterize *msmo1* expression in early zebrafish development, we used whole-mount *in situ* hybridization. Expression was first detected during early stages of somitogenesis in the yolk syncytial layer (YSL) (Fig. 2D), an extraembryonic cell that expresses several markers of the primitive liver (Li et al., 2007; Mudumana et al., 2004). Expression continued in the YSL at 3 days post-fertilization (dpf) (Fig. 2E), with a new domain appearing in the newly formed liver at 4 dpf and 5 dpf (Fig. 2F,F′). Consistent with the late onset of the *kol^nu7^* early phenotype, *msmo1* expression was not observed in skeletal elements during the first 5 days of development (Fig. 2E,F). Furthermore, there was no difference in the *in situ* signals for *msmo1* expression between wild-type and *kol^nu7^* siblings during the first 5 days of development (data not shown).

We next characterized *msmo1* expression during juvenile development by RNAscope *in situ* hybridization. In ∼2-month-old fish, SL ∼15 mm, *msmo1* expression was found within cartilaginous elements such as Meckel's cartilage, but not within intramembranous skeletal elements such as the dentary bone (Fig. S6H) (Bird and Mabee, 2003; Cubbage and Mabee, 1996). Importantly, expression of *msmo1* was seen within endochondral growth plates, specifically in the region corresponding to pre-hypertrophic chondrocytes (Kronenberg, 2003; LeClair et al., 2009) (see Fig. 5C,C′). In addition, we observed *msmo1* expression in the liver, kidney, intestine, brain, retina, spinal cord and skin (Fig. S6A-G). Comparison of *msmo1* expression in juvenile wild-type and *kol^nu7^* siblings revealed an undetectable level of *msmo1* expression within endochondral growth plates of *kol^nu7^* mutants (Fig. 5F,F′). Our findings support the notion that a deficit in *msmo1* expression, particularly in differentiating chondrocytes, underlies the *kol^nu7^* phenotype.

### The *kol^nu7^* mutation perturbs a *cis*-acting *msmo1* regulatory element

To confirm that loss of Msmo1 is solely responsible for the development of the *kol^nu7^* phenotype, we mutagenized the *msmo1* gene using CRISPR/Cas9 genome editing. We isolated an allele, *msmo1^nu81^*, which results in a 37 bp insertion (Fig. 1K) in the first coding exon, resulting in a frameshift and premature protein truncation. The *msmo1^nu81^* heterozygotes, similar to *kol^nu7/+^*, are phenotypically normal. Next, to test genetic complementation, we crossed *msmo1^nu81^* and *kol^nu7^* heterozygotes. Transheterozygote *kol^nu7/+^:msmo1^nu81/+^* appeared phenotypically normal until ∼5 weeks post-fertilization, after which they began to take on a *kol^nu7^*-like appearance and became morphologically indistinguishable from *kol^nu7^* mutants (Fig. 1I,J).

Our results suggest that the *kol^nu7^* mutation disrupts a regulatory element driving *msmo1* expression. We directly examined this possibility by taking advantage of a relatively large insertion in *msmo1^nu81^* (Fig. 1K) that allows us to compare allele-specific expression. Once again, we used total RNA isolated from the hypural complexes for semi-quantitative RT-PCR analysis (Fig. 1L). In contrast to *msmo1^nu81/+^*, in which both wild-type and mutant products can easily be identified after RT-PCR, the wild-type *msmo1* allele linked to the *kol^nu7^* locus is under-represented, strongly supporting the notion that the *kol^nu7^* mutation disrupts a *cis*-acting regulatory element*.* In summary, based on the results of our positional cloning, *in situ* hybridization and complementation testing, we concluded that the *kol^nu7^* mutant phenotype is a result of strongly reduced *msmo1* expression after loss of a positively acting regulatory element.

### Loss of Msmo1 function is lethal in zebrafish larvae

While *kol^nu7^* homozygote fish survive until adulthood, *msmo1^nu81^* homozygote mutants show a decrease in growth at 6 dpf (data not shown) and die by 9 dpf (Fig. 2A). Overexpression of wild-type *msmo1* driven by the *Tg(hsp70l*:*msmo1*:IRESnlsEGFP*)^nu99^* transgene was able to suppress the early lethality of *msmo1^nu81^* homozygotes (Fig. 2B), indicating that loss of Msmo1 is responsible for the death of the *msmo1^nu81^* mutants. Because Msmo1 activity plays a crucial role in cholesterol biosynthesis (Sharpe and Brown, 2013) (Fig. S1), we tested whether a cholesterol-enriched diet could extend the lifespan of *msmo1^nu81^* mutants. However, even a 4% cholesterol-enriched diet, shown to induce hypercholesterolemia in zebrafish (Stoletov et al., 2009), did not significantly improve mutants’ survival (Fig. 2C).

### Expression of *msmo1* in liver rescues *msmo1^nu81^* mutants and produces *kol^nu7^* phenotype

To study the role of Msmo1 in bone formation, we needed to suppress the early lethality of *msmo1^nu81^* mutants. We observed strong *msmo1* expression in the larval and juvenile liver (Fig. 2D-F′; Fig. S6A), an organ responsible for producing endogenous cholesterol (Turley, 2004). We therefore predicted that hepatic restoration of Msmo1 activity might rescue the *msmo1^nu81^* mutants, allowing us to study juvenile bone development in this genetic background. To test this prediction, we used two approaches. First, with the help of partial endoderm replacement, we created *msmo1^nu81^* mutants with chimeric endodermal organs. We pushed cells of the *Tg(ubi:Zebrabow-M)^a131^* (Pan et al., 2013) donor embryos to an endodermal fate by overexpressing *sox32* at the one-cell stage (Stafford et al., 2006). The red fluorescing donor cells were transplanted from high-stage donor embryos into shield-stage host embryos obtained from *msmo1^nu81^* heterozygote in-crosses (Fig. 2G). We analyzed the host fish at ∼5 weeks of development. Four of 15 transplanted fish, with significant contribution of donor cells, appeared to have a *kol^nu7^*-like appearance (Fig. 2H,I). Genotyping revealed that all of the *kol^nu7^*-like fish were chimeras with *msmo1^nu81^* mutants. In a parallel effort, we employed a transgenic approach to drive hepatic *msmo1* expression using the liver-specific regulatory element of the *fatty acid binding protein, liver basic* (*fabp10a*) gene (Her et al., 2003; Kwan et al., 2007) (Fig. 2J). In contrast to the transplantation experiments, the transgenic expression of *msmo1* was much more efficient in suppression of lethality, and close to Mendelian ratios of *msmo1^nu81^* mutants were found at 5 weeks in ∼700 fish studied from 13 separate crosses. Rescued mutants grew to adulthood and displayed a severe *kol^nu7^*-like phenotype (Fig. 2K,L). These results indicate that restoration of hepatic Msmo1 activity is sufficient for survival of *msmo1^nu81^* mutants beyond early larval stages. Interestingly, liver-derived cholesterol synthesis is unable to rescue the skeletal phenotype of *msmo1^nu81^* mutants, indicating a requirement for chondrocyte-specific sterol production.

### Loss of sterol synthesis partially suppresses *msmo1^nu81^* mutant phenotype

**Fig. 3.**
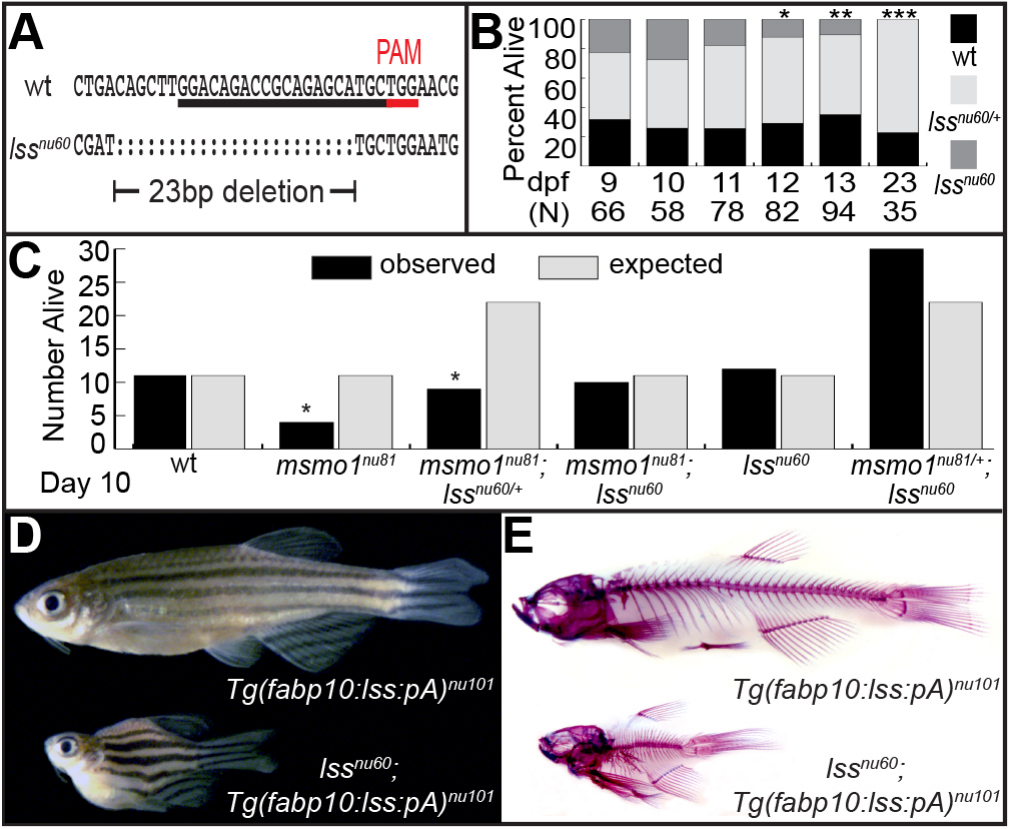
**Loss of Lss activity phenotype is epistatic to *msmo1^nu81^* mutation.** (A) The *lss^nu60^* allele. PAM, protospacer adjacent motif (underlined in red). (B) Survival analysis of clutches of *lss^nu60/+^* in-crosses, from 9 dpf to 23 dpf, reveals that most *lss^nu60^* mutants die by 12 dpf. [9 dpf wild type (wt; black) *n*=21, heterozygotes (het; pale gray) *n*=30, knockout mutants (KO; dark gray) *n*=15, *P*=0.4412; 10 dpf wt *n*=15, het *n*=27, KO *n*=16, *P*=0.8563; 11 dpf wt *n*=20, het *n*=44, KO *n*=14, *P*=0.3320; 12 dpf wt *n*=24, het *n*=48, KO *n*=10, *P=0.0277; 13 dpf wt *n*=33, het *n*=51, KO *n*=10, **P=0.0026; 23 dpf wt *n*=8, het *n*=27, KO *n*=0, ***P=0.0009.] (C) Survival analysis of clutches of *msmo1^nu81^*^/+^*;lss^nu60/+^* in-crosses reveals that loss of Lss activity increases survivability of *msmo1^nu81^* mutants at 10 dpf. [wt observed (O) *n*=11, expected (E) *n*=11; *msmo1^nu81^* O *n*=4, E *n*=11; *msmo1^nu81^;lss^nu60/+^* O *n*=9, E *n*=22; *msmo1^nu81^;lss^nu60^* O *n*=10, E *n*=11; *lss^nu60^* O *n*=12, E *n*=11; *msmo1^nu81/+^;lss^nu60^* O *n*=30, E *n*=22; *msmo1^nu81/+^;lss^nu60/+^* O *n*=40, E *n*=44; *msmo1^nu81/+^* O *n*=18, E *n*=22; *lss^nu60^*^/+^ O *n*=18, E *n*=22; *P=0.0431.] Two-tailed *P*-values were calculated using chi-squared test. (D,E) Suppression of early lethality of *lss^nu60^* mutants by liver-specific expression of *lss*. (D) Adult *Tg(fabp10:lss:pA)^nu101^;lss^nu60^* mutants phenocopy *kol^nu7^*. *Tg(fabp10:lss:pA)^nu101^ n*=12, *Tg(fabp10:lss:pA)^nu101^;lss^nu60^ n*=12. (E) Whole-mount skeletal preparations reveal gross malformations throughout *Tg(fabp10:lss:pA)^nu101^;lss^nu60^* craniofacial and axial skeleton, similar to those observed in *kol^nu7^*. *Tg(fabp10:lss:pA)^nu101^ n*=3, *Tg(fabp10:lss:pA)^nu101^;lss^nu60^ n*=5.

In most human syndromes resulting from mutations in genes involved in the post-squalene cholesterol biosynthesis pathway, a simultaneous decrease in cholesterol levels and an accumulation of sterol intermediates is observed (Porter and Herman, 2011). To elucidate the effects of cholesterol deprivation from sterol intermediates’ accumulation, we eliminated the activity of Lss. This enzyme catalyzes the cyclization of the first sterol in the cholesterol biosynthesis pathway (Huff and Telford, 2005) (Fig. S1). Mutation of the *lss* gene is predicted to result in a deficit of cholesterol biosynthesis, but not an accumulation of sterol intermediates (Sharpe and Brown, 2013). Using CRISPR/Cas9 genome editing, we generated the *lss^nu60^* allele, resulting in a 23 bp deletion (Fig. 3A), causing a frameshift and premature protein truncation. Heterozygote *lss^nu60^* fish are phenotypically normal and fertile. In contrast to *kol^nu7/+^:msmo1^nu81/+^* transheterozygotes, the study of over 180 fish from two separate crosses did not identify any signs of skeletal abnormalities in adult *kol^nu7/+^;lss^nu60/+^* animals (data not shown). This observation further supports the notion that a reduced level of Msmo1 activity, and not a general deficit in the cholesterol biosynthesis pathway, is responsible for the *kol^nu7^* phenotype.

The growth of *msmo1^nu81^* and *lss^nu60^* homozygote mutants lags behind that of wild-type siblings beginning at 6 dpf (data not shown). Unlike *msmo1^nu81^* mutants, *lss^nu60^* homozygotes live until 11 dpf (Fig. 3B). A measurement of total cholesterol levels at 8 dpf indicated a strong reduction in both mutants. Compared to wild-type siblings, total cholesterol levels of *lss^nu60^* homozygous mutants were 43% (s.d. 9.14, *P*=0.0011) of wild-type levels, whereas those of *msmo1^nu81^* homozygous mutants were 22% (s.d. 10.59, *P*=0.0006) of wild-type levels. To determine whether better survival is a result of a less-severe defect in cholesterol biosynthesis or an accumulation of sterol intermediates, we conducted an epistatic analysis. We found that double *lss^nu60^;msmo1^nu81^* mutants survived to 10 dpf in close to expected numbers, whereas most *msmo1^nu81^* single mutants were lost (Fig. 3C). Our results strongly suggest that an accumulation of sterol intermediates is responsible for the more-severe *msmo1^nu81^* phenotype, compared to that of *lss^nu60^* mutants.

### Loss of Msmo1 and Lss activity is associated with skeletal defects

To study juvenile bone development in the *lss^nu60^* knockout mutants, we again employed a transgenic approach to restore hepatic *lss* expression (Her et al., 2003; Kwan et al., 2007). Similar to the results described for the rescued *msmo1^nu81^* mutants, close to Mendelian ratios of rescued *lss^nu60^* were found at 5 weeks in ∼425 fish studied from nine separate crosses. Rescued mutants grew to adulthood and displayed a severe *kol^nu7^*-like phenotype (Fig. 3D). Skeletal staining revealed compressions of the axial skeleton due to vertebral column fusions and deformations in the craniofacial skeleton (Fig. 3E).

**Fig. 4.**
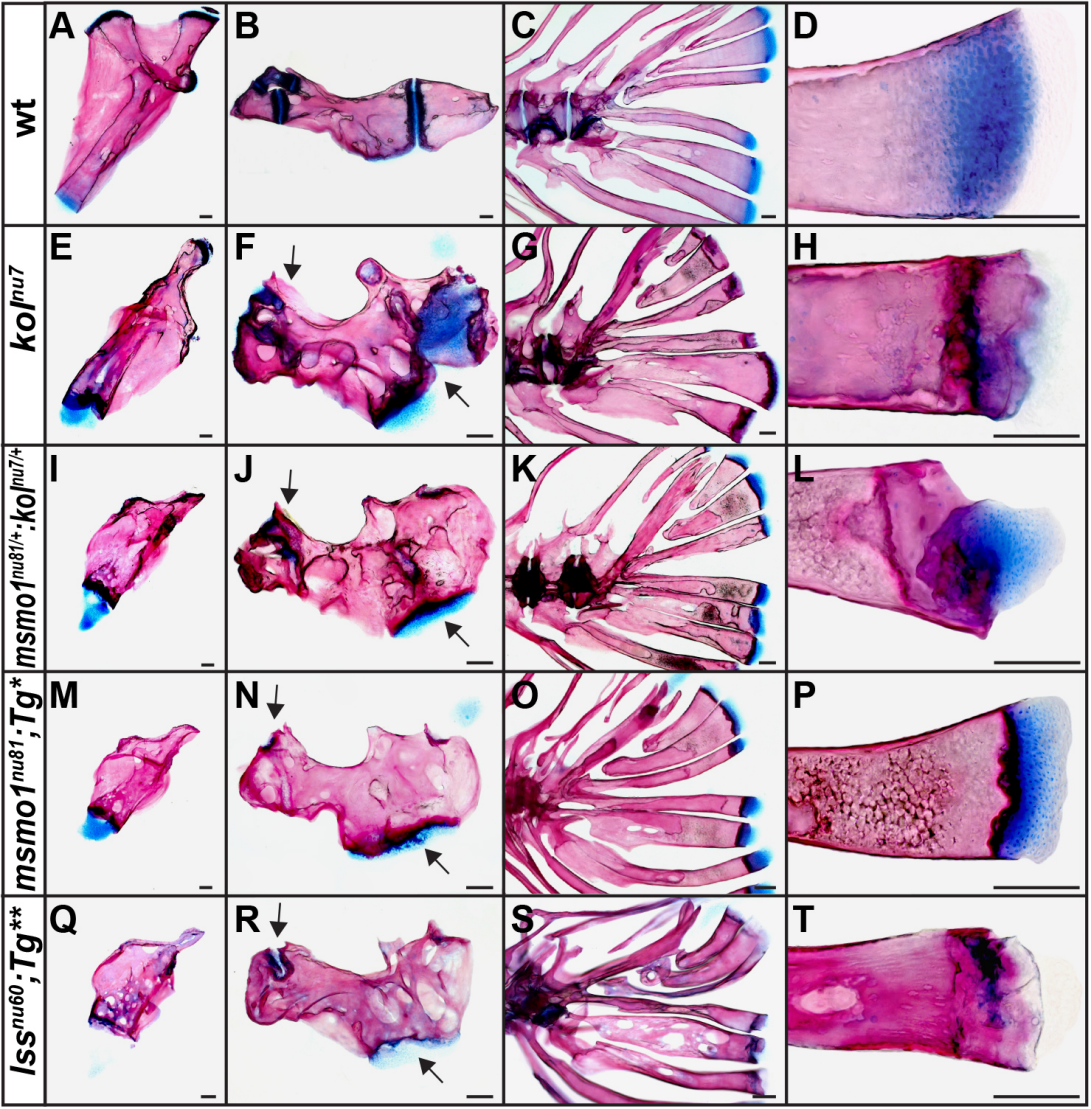
**Loss of Msmo1 and Lss activity is associated with abnormal endochondral bone formation.** (A-T) The hyomandibular (A,E,I,M,Q), ceratohyal (B,F,J,N,R) and hypural complex (C,D,G,H,K,L,O,P,S,T) were compared between wild type (A-D), *kol^nu7^* (E-H), *kol^nu7/+^:msmol^nu81/+^* (I-L), *Tg(fabp10a:msmo1:pA)^nu100^;msmo1^nu81^* (M-P) and *Tg(fabp10:lss:pA)^nu101^;lss^nu60^* (Q-T). (E-T) The endochondral bones of the mutants are compressed and irregularly shaped with ectopic ossification and fusions throughout the growth plates, marked with arrows. Images represent disarticulated whole-mount adult skeletal preparations at ∼4 months of age. Scale bars: 100μm. Tg*, *Tg(fabp10:msmo1:pA)^nu100^*; Tg**, *Tg(fabp10:lss:pA)^nu101^*. wt *n*=4, *kol^nu7^ n*=4, *kol^nu7/+^:msmol^nu81/+^ n*=4, *Tg(fabp10a:msmo1:pA)^nu100^;msmo1^nu81^ n*=4, *Tg(fabp10:lss:pA)^nu101^;lss^nu60^ n*=4.

To better understand the skeletal phenotype associated with loss of Msmo1 and Lss activity, we analyzed the skeletons of *kol^nu7^*, *kol^nu7/+^:msmo1^nu81/+^*, *Tg(fabp10a:msmo1:pA)^nu100^;msmo1^nu81^* and *Tg(fabp10:lss:pA)^nu101^;lss^nu60^* mutants. We collected mutant and wild-type siblings at 4 months and compared bone formation, focusing on selected bones formed by cartilage replacement mechanisms: the hyomandibular, ceratohyal and hypural complex (Bird and Mabee, 2003; Cubbage and Mabee, 1996) (Fig. 4). The bones of mutants and transheterozygotes were irregularly shaped (Fig. 4A,E,I,M,Q) with abnormal stratification of the growth plates (Fig. 4D,H,L,P,T). The persistent cartilage bands separating individual bones (Fig. 4B), which normally never fuse in zebrafish (LeClair et al., 2009), were often closed as a result of ectopic ossification (Fig. 4F,J,N,R). The endochondral bones of the *kol^nu7/+^:msmo1^nu81/+^* (Fig. 4I-L), *Tg(fabp10a:msmo1:pA)^nu100^;msmo1^nu81^* (Fig. 4M-P) and *Tg(fabp10:lss:pA)^nu101^;lss^nu60^* mutants (Fig. 4Q-T) phenocopied those of the *kol^nu7^* mutant (Fig. 4E-H), indicating that the loss of Msmo1 and Lss activity within cartilage-derived elements produces severely deformed, compressed endochondral bones with frequent ectopic ossification of the growth plate.

### Msmo1 and Lss activity in the growth plate is needed for normal chondrocyte differentiation

**Fig. 5.**
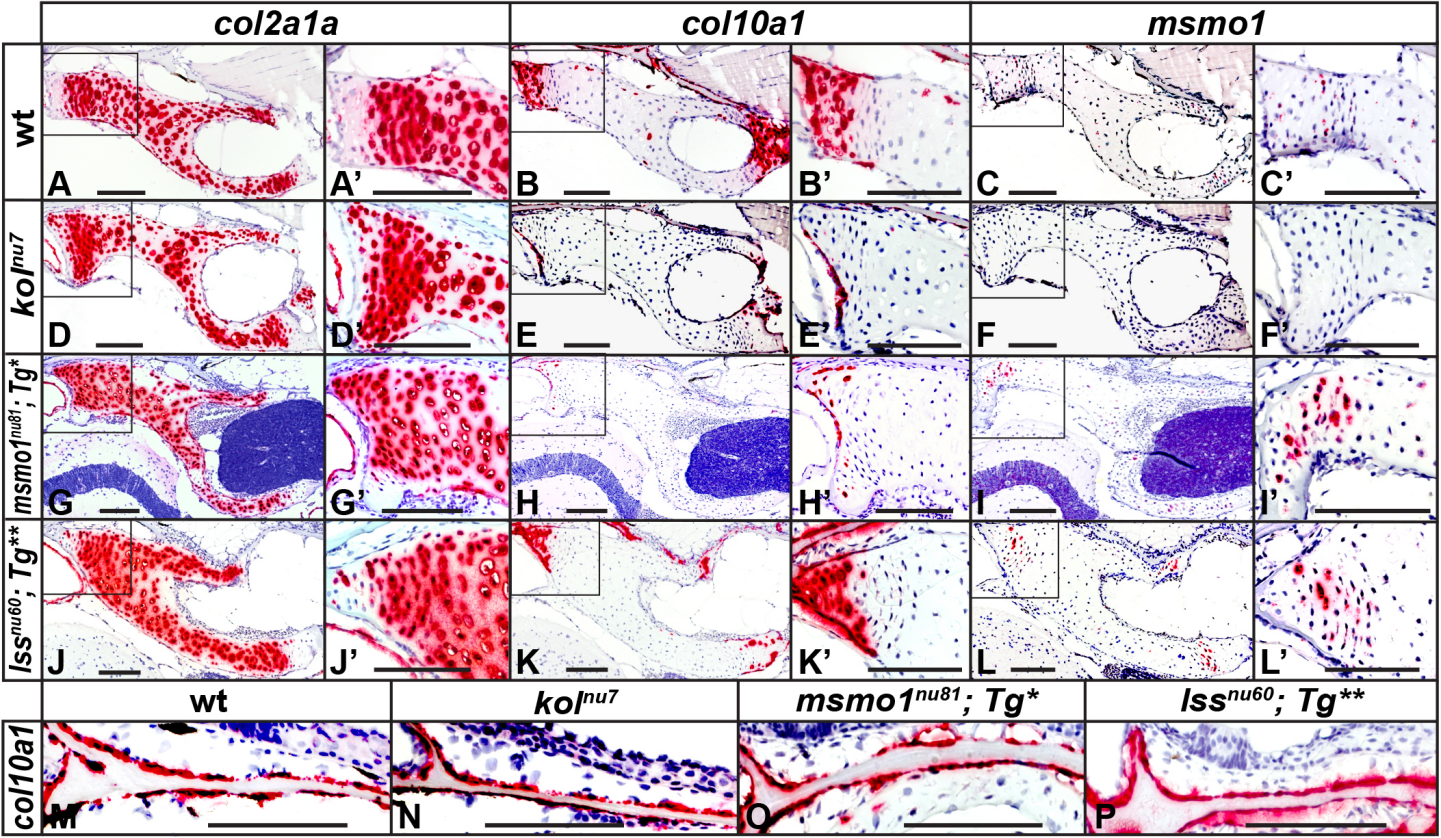
**Loss of Msmo1 and Lss activities disrupts growth plate patterning.** (A-P) Expression domains of *col2a1a* (A,D,G,J), *col10a1a* (B,E,H,K,M-P) and *msmo1* (C,F,I,L) were analyzed within growth plates of wild type (A-C), *kol^nu7^* (D-F), *Tg(fabp10a:msmo1:pA)^nu100^;msmo1^nu81^* (G-I) and *Tg(fabp10:lss:pA)^nu101^;lss^nu60^* (J-L) using RNAscope *in situ* hybridization. (A) Strong *col2a1a* expression is observed in round and stacked chondrocytes in wild type, corresponding to presumed resting and proliferating chondrocytes. (B) Expression of *col10a1a* in wild type is observed complementary to *col2a1a* expression in presumed hypertrophic chondrocytes. The expression of *col2a1a* is expanded in *kol^nu7^* (D) and *Tg(fabp10a:msmo1:pA)^nu100^;msmo1^nu81^* (G) growth plates, with a near-complete loss of *col10a1a* expression in *kol^nu7^* (E) and *Tg(fabp10a:msmo1:pA)^nu100^;msmo1^nu81^* (H). Expression of *col2a1a* (J) and *col10a1a* (K) in *Tg(fabp10:lss:pA)^nu101^;lss^nu60^* appears less affected when compared to *kol^nu^*^7^(D,E) and *Tg(fabp10a:msmo1:pA)^nu100^;msmo1^nu81^* (G,H). Expression of *msmo1* is detected primarily in pre-hypertrophic chondrocytes of wild type (C); *msmo1* expression is undetectable in *kol^nu7^* growth plates (F), but is upregulated in transgenically rescued *msmo1^nu81^* (I) and *lss^nu60^* (L) mutants. (M-P) Expression of *col10a1a* in osteoblasts is unchanged between wild type and mutants. Images represent paraffin sections of the pterotic at 2 months of age (∼15 mm SL). Boxed areas in A-L are shown at higher magnification in A′-L′. Scale bars: 100 μm. Tg*, *Tg(fabp10:msmo1:pA)^nu100^*; Tg**, *Tg(fabp10:lss:pA)^nu101^*. wt *n*=6, *kol^nu7^ n*=6, *Tg(fabp10a:msmo1:pA)^nu100^;msmo1^nu81^ n*=3, *Tg(fabp10:lss:pA)^nu101^;lss^nu60^ n*=3.

To examine changes in growth plate patterning after loss of Msmo1 and Lss activity, we used RNAscope *in situ* hybridization to analyze the gene expression of key markers in the growth plate of the pterotic bone, an element of the chondocranium (Cubbage and Mabee, 1996). We focused on *collagen type II, alpha 1* (*col2a1a*), a marker of resting and proliferating chondrocytes (Dale and Topczewski, 2011; Zhao et al., 1997), and *collagen type 10, alpha 1a* (*col10a1a*), a marker of hypertrophic chondrocytes (Dale and Topczewski, 2011; Eames et al., 2012; Topczewska et al., 2016) and osteoblasts (Avaron et al., 2006; Eames et al., 2012; Kim et al., 2013). We analyzed wild-type, *kol^nu7^*, *Tg(fabp10a:msmo1:pA)^nu100^;msmo1^nu81^* and *Tg(fabp10:lss:pA)^nu101^;lss^nu60^* fish at ∼2 months of age, when wild-type fish had an SL of ∼15 mm. In wild-type animals, strong *col2a1a* expression was observed in round and stacked chondrocytes (Fig. 5A,A′), corresponding to presumed resting and proliferative zones of the growth plate. In a complementary pattern, *col10a1a* was detected in the presumed hypertrophic chondrocytes (Fig. 5B,B′). In the *kol^nu7^* and *Tg(fabp10a:msmo1:pA)^nu100^;msmo1^nu81^* mutants, *col2a1a* expression is expanded (Fig. 5D,D′,G,G′) and accompanied by near complete loss of *col10a1a* expression (Fig. 5E,E′,H,H′). Although a similar expansion of the of *col2a1a* domain was observed in *Tg(fabp10:lss:pA)^nu101^;lss^nu60^* mutants (Fig. 5J,J′), *col10a1a*-positive chondrocytes were present in all growth plates studied (Fig. 5K,K′). These results indicate that although Lss activity is needed for proper growth plate development, loss of Msmo1 activity has a more-severe effect on chondrocyte differentiation. Importantly, the osteoblast expression of *col10a1a* in all mutants was similar to that in wild type (Fig. 5M-P), indicating a defect specific to chondrocyte differentiation. Interestingly, the domain of *msmo1* expression in wild-type growth plates is localized to the transition zone from the columnar to hypertrophic chondrocytes (Fig. 5C,C′). This domain is absent in *kol^nu7^* mutants (Fig. 5F,F′), consistent with the proposed regulatory nature of the mutation. In contrast, the *msmo1* expression domain is expanded in the *Tg(fabp10a:msmo1:pA)^nu100^;msmo1^nu81^* and *Tg(fabp10:lss:pA)^nu101^;lss^nu60^* mutants (Fig. 5I,I′,L,L′), suggesting de-repression of the pathway. Based on these results, we conclude that, during normal development, *msmo1* is transiently upregulated in pre-hypertrophic chondrocytes, and loss of the ability to synthesize cholesterol impedes chondrocyte hypertrophic differentiation.

### Loss of Msmo1 and Lss activity within growth plates does not result in loss of Ihh signaling

**Fig. 6.**
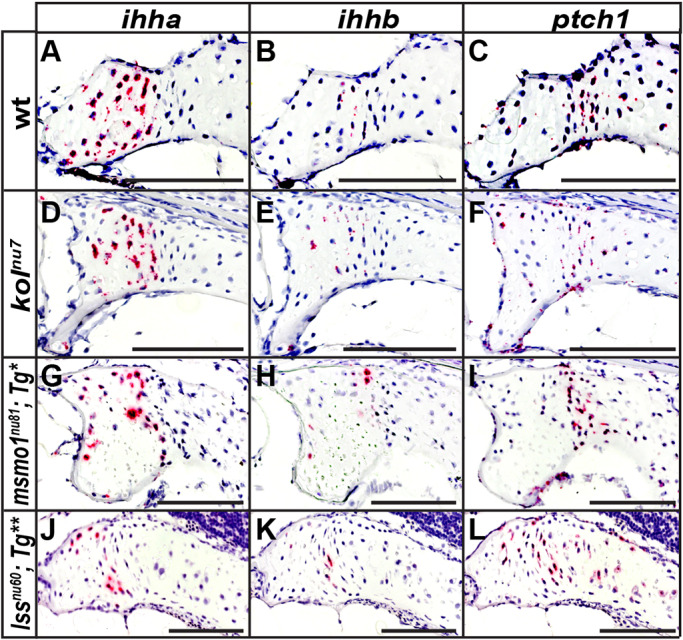
**Ihh signaling within endochondral growth plates is present despite loss of Msmo1 and Lss activity.** (A-L) Expression domains of *ihha* (A,D,G,J), *ihhb* (B,E,H,K) and *ptch1* (C,F,I,L) were analyzed within growth plates of wild type (A-C), *kol^nu7^* (D-F), *Tg(fabp10a:msmo1:pA)^nu100^;msmo1^nu81^* (G-I) and *Tg(fabp10:lss:pA)^nu101^;lss^nu60^* (J-L) using RNAscope *in situ* hybridization. (A-C) In wild type, expression of *ihha* and *ihhb* is observed in the pre-hypertrophic chondrocyte zone, with *ptch1* expression seen to either side of these domains. (D-L) Expression of *ihha*, *ihhb* and *ptch1* is present within mutant growth plates and similar to domains observed in wild type. Images represent paraffin sections of the pterotic at 2 months of age (∼15 mm SL). Scale bars: 100 μm. Tg*, *Tg(fabp10:msmo1:pA)^nu100^*; Tg**, *Tg(fabp10:lss:pA)^nu101^*. wt *n*=3, *kol^nu7^ n*=3, *kol^nu7/+^:msmol^nu81/+^ n*=3, *Tg(fabp10a:msmo1:pA)^nu100^;msmo1^nu81^ n*=3, *Tg(fabp10:lss:pA)^nu101^;lss^nu60^ n*=3.

Ihh is a key ligand in cartilage and bone development. Ihh is synthesized by pre-hypertrophic chondrocytes and stimulates chondrocyte proliferation and hypertrophy, as well as osteoblast differentiation (Hammond and Schulte-Merker, 2009; St-Jacques et al., 1999; Vortkamp et al., 1996). Given the critical role of cholesterol in the post-translational Hedgehog (Hh) modification (Chen et al., 2011; Porter et al., 1995), we investigated what potential effect loss of Msmo1 and Lss activity may have on Ihh signaling within growth plates. Zebrafish have two Ihh paralogs, *ihha* and *ihhb*, with partially overlapping expression domains (Avaron et al., 2006). The receptor *patched 1* (*ptch1*)*,* a transcriptional target of Hh signaling, is expressed in chondrocytes and the perichondrium of endochondral bone (Felber et al., 2011; Hammond and Schulte-Merker, 2009; St-Jacques et al., 1999; Vortkamp et al., 1996). We analyzed expression of *ihha*, *ihhb* and *ptch1* by RNAscope *in situ* hybridization using the same stages and anatomical location as described above. In wild-type fish, a broader *ihha* domain of expression in the hypertrophic chondrocytes zone and a more restricted *ihhb* domain of expression in mostly the pre-hypertrophic chondrocytes zone (Fig. 6A,B) was observed. At the same time, strong *ptch1* expression was observed in the adjacent columnar zone and in individual hypertrophic chondrocytes (Fig. 6C). Expression of *ihha* and *ihhb* was still observed in growth plates of *kol^nu7^*, *Tg(fabp10a:msmo1:pA)^nu100^;msmo1^nu81^* and *Tg(fabp10:lss:pA)^nu101^;lss^nu60^* mutants (Fig. 6D,E,G,H,J,K). Importantly, *ptch1* expression was also detected in all mutant growth plates (Fig. 6F,I,L), indicating that loss of cholesterol biosynthesis in the mutants does not abolish Ihh signaling activity.

## DISCUSSION

### Zebrafish post-squalene mutants show phenotypic similarities to human CDP

**Fig. 7.**
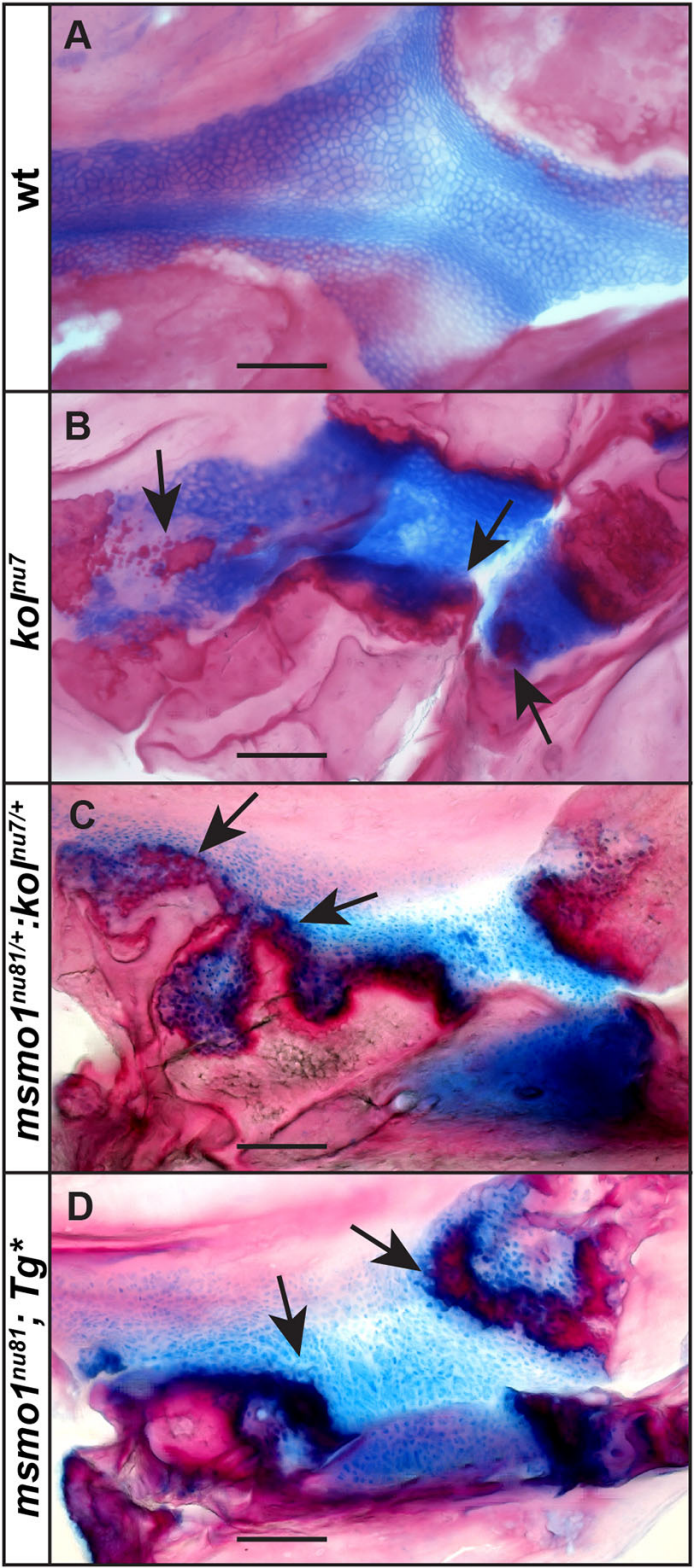
**New zebrafish models for CDP.** (A-D) Abnormal ossification, marked with arrows, within endochondral growth plates of *kol^nu7^* (B), *kol^nu7/+^:msmol^nu81/+^* (C) and *Tg(fabp10a:msmo1:pA)^nu100^;msmo1^nu81^* (D) mutant phenotypes resembles defects observed in patients with chondrodysplasia punctata. Images represent lateral views of palatoquadrate growth plates of craniofacial bones from disarticulated whole-mount adult skeletal preparations at ∼4 months of age. Scale bars: 100 μm. Tg*, *Tg(fabp10:msmo1:pA)^nu100^*. wt *n*=4, *kol^nu7^ n*=6, *kol^nu7/+^:msmol^nu81/+^ n*=4, *Tg(fabp10a:msmo1:pA)^nu100^;msmo1^nu81^ n*=4.

Many post-squalene pathway disorders with documented CDP result from partial loss-of-function mutations, such as mutations in the NAD(P)-dependent steroid dehydrogenase-like (*NSDHL*) gene (Fig. S1), which result in congenital hemidysplasia with ichthyosiform erythroderma and limb defects (CHILD) syndrome (Bornholdt et al., 2005; Mi et al., 2015; Herman and Kratz, 2012), and mutations in the emopamil binding protein (*EBP*) gene (Fig. S1), which result in X-linked dominant chondrodysplasia punctata (CDPX2), or Conradi-Hunermann-Happle syndrome (Cañueto et al., 2014; Herman and Kratz, 2012). Similarly, the *kol^nu7^*, *kol^nu7/+^:msmo1^nu81/+^* and *Tg(fabp10a:msmo1:pA)^nu100^;msmo1^nu81^* mutants result from reduction and/or absence of Msmo1 activity within skeletal elements and display ectopic ossification within growth plates similar to that seen in CDP (Figs 4 and 7) (Cañueto et al., 2014; Herman and Kratz, 2012; Rossi et al., 2015). As such, these mutants could prove helpful as animal models to help understand the mechanisms behind the development, and potential treatment, of CDP.

### Sterol intermediates may play a role in *msmo1^nu81^* mutant phenotype

A lack of cholesterol alone does not explain all of the defects observed in post-squalene cholesterol biosynthesis disorders, as many patients present with normal cholesterol levels (Porter, 2008; Porter and Herman, 2011). Hypomorphic mutations in *MSMO1* result in the rare autosomal recessive disorder C4 methyloxidase-like deficiency (He et al., 2011). Since patients may not present with decreased cholesterol levels, the definitive test for this disorder is an accumulation of C4 sterol intermediates (Frisso et al., 2017; He et al., 2011, 2014; Herman and Kratz, 2012; Rossi et al., 2015). Similarly, patients with Smith-Lemli-Opitz syndrome, which results from mutations in the 7-dehydrocholesterol reductase (*DHCR7*) gene (Fig. S1), and CDPX2, a result of mutations in the *EBP* gene (Fig. S1), often present with normal cholesterol levels and elevated levels of sterol intermediates (Braverman et al., 1999; Porter, 2008; Porter and Herman, 2011; Wassif et al., 1998).

Treatment regimens for many post-squalene pathway disorders may include dietary cholesterol supplementation and statin drug therapy (Porter and Herman, 2011). Statins act by impeding cholesterol synthesis through inhibition of HMG-CoA reductase (Sharpe and Brown, 2013) (Fig. S1). It is thought that statins may help prevent a buildup of potentially toxic sterol intermediates. Similar to statin treatment, inactivation of LSS activity impedes cholesterol synthesis and prevents sterol intermediate accumulation. But, unlike statin treatment, inactivation of LSS does not affect isoprenoid synthesis (Fig. S1). The protective effect provided by loss of Lss function in the *msmo1^nu81^* mutants supports this prediction. Both *msmo1^nu81^* and *lss^nu60^* mutants are deficient in cholesterol biosynthesis, and both are characterized by progressive growth retardation and lethality soon after depletion of maternally deposited lipid and cholesterol-rich yolk (Miyares et al., 2014; Quinlivan and Farber, 2017) (Figs 2A and 3B). However, the *lss^nu60^* mutants live 3 days longer (Fig. 3B) than *msmo1^nu81^* mutants (Fig. 2A), suggesting that toxic sterol intermediates may contribute to the death of *msmo1^nu81^* mutants. Importantly, the earlier lethality of the *msmo1^nu81^* mutants can be suppressed by a loss of Lss activity (Fig. 3C), indicating a protective effect through inhibition of sterol intermediate formation.

Correspondingly, a more-severe patterning defect is observed in *Tg(fabp10a:msmo1:pA)^nu100^;msmo1^nu81^* mutant growth plates compared to *Tg(fabp10:lss:pA)^nu101^;lss^nu60^* mutants. In particular, the *col10a1a* expression in *kol^nu7^* (Fig. 5E,E′) and *Tg(fabp10a:msmo1:pA)^nu100^;msmo1^nu81^* (Fig. 5H,H′) mutant growth plates is nearly absent. At the same time, the *col10a1a*-positive domain is reduced but not completely eliminated in *Tg(fabp10:lss:pA)^nu101^;lss^nu60^* mutants (Fig. 5K,K′). Our results suggest that both Msmo1 and Lss activity are needed for proper growth plate patterning and efficient chondrocyte differentiation. However, blockage of the pathway at the C4 demethylation step is associated with a more-severe defect, indicating a role for sterol intermediates, in addition to cholesterol depletion, in growth plate patterning and chondrocyte differentiation. Our findings point to Lss as an interesting target to mitigate some symptoms associated with disorders of the post-squalene cholesterol biosynthesis pathway without disrupting isoprenol-dependent processes.

### Growth plates of *kol^nu7^* and *Tg(fabp10a:msmo1:pA)^nu100^;msmo1^nu81^* mutants show distinct phenotype

The growth plates of *kol^nu7^* and *Tg(fabp10a:msmo1:pA)^nu100^;msmo1^nu81^* are unique in that they display an enhanced and ectopic ossification phenotype (Fig. 4E-H,M-P), yet patterning of the growth plates revealed loss of *col10a1a* expression and absence of the presumptive hypertrophic chondrocyte zone (Fig. 5E,E′,H,H′). Interestingly, Ihh genes are still expressed in the mutants, suggesting that the transition from proliferating to pre-hypertrophic zone is not inhibited. The enhanced ossification phenotype seen in *kol^nu7^* and *Tg(fabp10a:msmo1:pA)^nu100^;msmo1^nu81^* mutants appears similar to that in zebrafish *cyp26b1* mutants, but these mutants do not show a loss of *col10a1a* expression in endochondral bones (Laue et al., 2008; Spoorendonk et al., 2008). The unique phenotype of *kol^nu7^* and *Tg(fabp10a:msmo1:pA)^nu100^;msmo1^nu81^* mutants most closely resembles Sox9 inactivation in mouse growth plates (Dy et al., 2012; Ikegami et al., 2011). Sox9 is a master regulator of chondrocyte differentiation and survival (Lefebvre et al., 2019), indicating that Msmo1 activity within pre-hypertrophic chondrocytes might play a role in controlling cell hypertrophy and chondrocyte differentiation within the growth plate.

These results indicate that cholesterol depletion, and possibly accumulation of sterol intermediates, within mutant chondrocytes exiting from the proliferation zone may prevent normal differentiation and proper growth plate patterning, leading to an accelerated ossification. Intriguingly, CDP is observed in patients with a deficit in the initial steps of the post-squalene cholesterol biosynthesis pathway (Rossi et al., 2015), pointing to a potential active role of sterol intermediates in the first steps of biosynthesis in bone pathology. Recently, sterol intermediates were implicated in interactions with numerous signaling pathways by acting as ligands for LXRs (also known as NR1Hs), RORγ (also known as RORC) and EGFR (Gabitova et al., 2015; Hu et al., 2015; Santori et al., 2015; Sukhanova et al., 2013; Urlep et al., 2017; Yang et al., 2006). These data highlight the importance of sterol intermediates in the cholesterol biosynthesis pathway and the role these intermediates may play in skeletal dysplasia.

### Msmo1 and Lss activity in growth plates is not essential for Ihh signaling

It is currently proposed that defects observed in disorders of the post-squalene pathway are due to two main mechanisms: abnormal Hh signaling and accumulation of sterol intermediates (Porter and Herman, 2011). Cholesterol modification of the Hh protein is necessary for proper Hh signaling and influences the spread of the morphogen through tissue (Briscoe and Thérond, 2013; Zeng, 2001; Zeng et al., 2001). Recently, it has been shown that cholesterol interacts with Smoothened (SMO) and the inability of SMO to bind cholesterol negatively affects Hh signaling (Byrne et al., 2016).

The role of Hh signaling in skeletogenesis is well known, with Shh playing a pivotal role in early patterning and Ihh acting later during bone formation (Yang et al., 2015). Thus, defects resulting in impaired cholesterol biosynthesis can affect Hh signaling, and many bone defects seen in cholesterol biosynthesis disorders, such as polydactyly, syndactyly and cleft palate, are hallmarks of abnormal Shh signaling (Briscoe and Thérond, 2013; Cooper et al., 2003; Gao et al., 2009; Hui, 1993; Hui and Joyner, 1993). It is interesting to note that although the *kol^nu7^*, *msmo1^nu81^* and *lss^nu60^* mutations negatively affect cholesterol synthesis, these mutants do not display the classical patterning defects associated with defective Shh signaling (Eberhart et al., 2006; Schwend and Ahlgren, 2009; Wada et al., 2005) (Fig. S4). This is likely to be caused by maternally deposited cholesterol within the yolk of the zebrafish that allows for normal embryonic and early larval development (Miyares et al., 2014; Quinlivan and Farber, 2017). Zebrafish have a large yolk that is slowly absorbed over the first 5 days of development (Miyares et al., 2014; Quinlivan and Farber, 2017). Therefore, zebrafish larvae do not require an endogenous biosynthesis of cholesterol until ∼6 dpf.

Although juvenile *kol^nu7^* mutants display an absence of *msmo1* expression in growth plates (Fig. 5F,F′), Ihh signaling within *kol^nu7^* growth plates is not lost (Fig. 6D-F). Similarly, juvenile *Tg(fabp10a:msmo1:pA)^nu100^;msmo1^nu81^* and *Tg(fabp10:lss:pA)^nu101^;lss^nu60^* mutants exhibit loss of Msmo1 and Lss activity, respectively, yet expression of Ihh ligands and their transcriptional target *ptch1* can be detected (Fig. 6G-L). These results suggest that the level of cholesterol in chondrocytes depleted of Msmo1 and Lss activity is sufficient to maintain Hh signaling.

### The *kol^nu7^* mutant highlights the significance of *cis*-acting regulatory mutations in disease

The importance of regulatory elements in human health and disease has been well recognized (Venter et al., 2001; Wray, 2007)*.* With protein-coding exons making up less than 2% of the human genome (Venter et al., 2001), *cis*-regulatory elements are now believed to be more important than gene-encoding sequences in determining human traits and susceptibility to disease (Niu et al., 2018). In fact, many skeletal malformations are the result of mutations in regulatory elements that control expression of genes such as bone morphogenetic protein 2 (*BMP2*) (Dathe et al., 2009), *SOX9* (Kurth et al., 2009), distal-less homeobox 5/6 (*DLX5/6*) (Kouwenhoven et al., 2010) and *SHH* (Albuisson et al., 2011; Wieczorek et al., 2010). Given the importance of regulatory elements in disease, the identification of new *cis*-regulatory mutations and the genes they regulate is crucial. The region harboring the *kol^nu7^* locus and *msmo1* gene is highly syntenic with a corresponding region of human chromosome 4 (data not shown), raising the possibility that similar long-distance regulation mechanisms operate in higher vertebrates.

### Mutations in *msmo1* and *lss* reveal an important role of cholesterol biosynthesis genes in bone development

Previously, very little was known of the role *MSMO1* and *LSS* played in bone development. Of the five reported cases of C4 methyloxidase-like deficiency, symptoms reported include cataracts, intellectual delays, skin abnormalities, immune dysfunction, shortened stature and microcephaly (Frisso et al., 2017; He et al., 2011, 2014; Herman and Kratz, 2012; Rossi et al., 2015). Of the seven published articles on *MSMO1* gene function, none show its role in bone development. Similarly, very few published articles show a link between *LSS* gene function and bone development. In humans, hypomorphic mutations in *LSS* result in congenital cataracts (Chen and Liu, 2017; Zhao et al., 2015), hypotrichosis simplex (Romano et al., 2018) and alopecia with mental retardation syndrome (Besnard et al., 2019).

Here, for the first time, we show that *msmo1* and *lss* are needed for proper chondrocyte differentiation, growth plate formation and endochondral ossification. To our knowledge, the *msmo1^nu81^* and *lss^nu60^* mutants reported here are the first-described loss-of-function fish mutants in Msmo1 and Lss, respectively. The *kol^nu7^* and *msmo1^nu81^* mutants are the first-reported animal models for the human disorder C4 methyloxidase-like deficiency. These mutants, along with the stable transgenic mutant lines *Tg(fabp10a:msmo1:pA)^nu100^;msmo1^nu81^* and *Tg(fabp10:lss:pA)^nu101^;lss^nu60^* described above, provide essential tools to further elucidate the roles of *msmo1* and *lss* in skeletal dysplasia. Zebrafish are an excellent tool for pre-clinical drug testing (MacRae and Peterson, 2015; Tan and Zon, 2011); thus, the lines presented here may provide invaluable insight into treatment options for those suffering from C4 methyloxidase-like deficiency, as well as other disorders of the post-squalene cholesterol biosynthesis pathway.

## MATERIALS AND METHODS

### Positional cloning

The *kol^nu7^* mutation was localized to zebrafish chromosome 1 using a genetic linkage map of sequence-length polymorphism markers (Knapik et al., 1998). Additional closely linked markers, based on either restriction enzyme polymorphism or sequencing for single-nucleotide polymorphism were used to narrow down the *kol^nu7^* location to a ∼457 kb critical region (Fig. 1C).

### MultAlin analysis

All protein sequences were obtained from UniProtKB (UniProt, 2019): UGT8 *Homo sapiens* (Entry ID: Q16880), UGT8 *Gallus gallus* (Entry ID: *F*1NH08) and Ugt8 *Danio rerio* (Entry ID: Q1ECX6). These sequences were then aligned using MultAlin (Corpet, 1988), using an absolute scoring method and Blosum62 symbol comparison table with a gap opening default value of 12 and gap extension default value of 2.

### RNA extraction

To extract bone-enriched total RNA, the hypural complexes from *kol^nu7^* and wild-type siblings were dissected after the SL for each animal was recorded. Each hypural complex was placed in a 1.5-ml RINO Navy Bead tube (RINO BulletBlender Navy Bead Lysis kit; Next Advance, Troy, NY, USA) with 600 μl TRIzol (Invitrogen). Tissue was homogenized using the BeadBug Microtube Homogenizer (Benchmark Scientific, Sayreville, NJ, USA). Samples were extracted using five cycles of 1 min homogenization followed by 1 min cooling in a wet ice bath. RNA was purified from the TRIzol solution using Directzol RNA Minipreps (Zymo Research, Irvine, CA, USA).

### Quantitative RT-PCR

All assays were performed using an EXPRESS One-Step Superscript qRT-PCR Kit, with premixed ROX (#11791200; Thermo Fisher Scientific, Waltham, MA, USA), Applied Biosystems MicroAmp Fast Optical reaction plates and 8-cap strips (Thermo Fisher Scientific) on an Applied Biosystems StepOnePlus Real-Time PCR System. For each reaction, 8 ng total RNA was used. All assays consisted of three technical replicates and a minimum of two biological replicates. The following Made to Order Taqman probes (Thermo Fisher Scientific) were used: *mettl14* (Assay ID: Dr03438758_g1), *tll1* (Assay ID: Dr03138096_m1), *cpe* (Assay ID: Dr03188311_s1), *grhprb* (Assay ID: Dr03088390_g1), *ugt8* (Assay ID: Dr03427025) *msmo1* (Assay ID: Dr03133463_m1) and *eef1a1l1* (Assay ID: Dr03432748_m1). In addition, the following Custom Plus Taqman probes (Thermo Fisher Scientific) were designed and used: *ndst3* (Assay ID: AJY9Y10), *spock3* (Assay ID: AJ20TKF), *prss12* (Assay ID: AJ5IPWS), *apbb2* (Assay ID: *AJT96CS)* and *uba6* (Assay ID: AJPADNW). The efficiency of each probe was tested prior to experimentation using the following equation: PCR efficiency (%)=(10^(–1/S)^ – 1)×100, where S=slope of the standardized curve.

### Semi-quantitative RT-PCR

All assays were performed using a Superscript III One-Step RT-PCR System with Platinum Taq DNA Polymerase. For each reaction, 30 ng RNA was used. The wild-type, *kol^nu7^* and *msmo1^nu81^* alleles were amplified using the primers 5′-TGGTCTTATGTGAACTTTCTTTACA-3′ and 5′- GATAAATCCAGGCAGGCAGA-3′. The PCR products were separated using a 3.5% Metaphor agarose (Lonza) gel. All assays consisted of three biological replicates.

### CRISPR/Cas9 genome editing

Genome editing was performed as previously described (Hwang et al., 2013; Jao et al., 2013). We used linearized plasmid pCS2-nCas9n (Addgene plasmid #47929) (Jao et al., 2013) as a template for *Cas9* mRNA synthesis (mMessage mMachine; Thermo Fisher Scientific). The guide RNA (gRNA) template was made by PCR and RNA was synthesized using a MEGAshortscript T7 kit (Thermo Fisher Scientific). In all cases, 30-40 pg gRNA was co-injected with 150 pg *Cas9* mRNA per embryo at one-cell stage. Indels were identified by loss of restriction enzyme sites. The G0 founders positive for mutation were outcrossed with wild-type fish to produce *F*1 heterozygotes, which were then analyzed using sequencing to identify the mutations. Individuals carrying desirable, identified mutations were outcrossed with wild type to generate an *F*2 heterozygous family.

### High-cholesterol diet (HCD)

Beginning at 5 dpf, larvae were fed a HCD similar to that previously described (Stoletov et al., 2009). Fish were raised in 450 ml system water, 6 parts per thousand (ppt) salinity, in 2.8-l tanks, and fed 15 mg of either a normal diet or HCD twice daily. Water was changed daily. Normal (0.12% cholesterol) and HCD (4% cholesterol) was provided by the Department of Biology, University of Alabama at Birmingham, Birmingham, AL, USA (Watts et al., 2012).

### Total cholesterol measurement

Mutant clutches were collected in Petri dishes filled with egg water at 0 dpf. Embryos were placed in a 28**°**C incubator and fasted until collection at 8 dpf when they were snap frozen. An eyeball of each larva was removed for genotyping purposes. Once genotyping results were confirmed, individual larvae were homogenized in 200 µl extraction buffer chloroform:isopropanol:IGEPAL CA-630 (7:11:0.1) using a micro-tube homogenizer. The extraction procedure and total cholesterol measurements were carried out using a BioVision Total Cholesterol and Cholesteryl Ester Colorimetric/Fluorometric Assay Kit (#K603-100), as per the manufacturer’s recommendations, with 25 µl extracted larva sample used per assay, and 25 µl Reaction Mix or 25 µl Background Control Mix used per well. All assays consisted of three technical sample replicates, three technical background control replicates and three biological replicates. Fluorescence was detected using a CLARIOstar Microplate Reader (BMG Labtech, Ortenberg, Germany). Mean relative fluorescence units (RFUs) for each sample were calculated by subtracting the mean background control value from the mean sample value. From these data, the mean RFU for each group was calculated. Wild-type mean RFU=206081, s.d. 8150, s.e.m. 4705, *n*=3; *msmo1^nu81^* mean RFU=45401, s.d. 26629, s.e.m. 15374, *n*=3 (*P*=0.0006); *lss^nu60^* mean RFU=89447, s.d. 22756, s.e.m. 13138, *n*=3 (*P*=0.0011). Mutant RFU levels were compared to wild-type RFU levels to determine the percentage of total cholesterol levels. *P*-values were calculated using unpaired Student's *t*-test.

### Embryonic cell transplantation

Transplantation was performed as previously described (Ho and Kane, 1990; Stafford et al., 2006). Wild-type donor embryos were co-injected at one- to two-cell stage with 20 ng/μl *sox32* mRNA and 40 kD fluorescein dextran to allow for visualization of donor cells. Approximately 20-30 cells from high-stage donors [∼3 h post-fertilization (hpf)] were distributed along the blastoderm margin of shield-stage (∼6 hpf) hosts generated form a *msmo1^nu81^* heterozygote in-cross. Then, 3 dpf embryos with a significant number of transplanted cells located in the intestines were selected for growth. At ∼6 weeks of age, fish were photographed using a Zeiss V-8 stereomicroscope equipped with a Zeiss Axiocam camera. To detect *Tg(ubi:Zebrabow-M)^a131^* donor cells, a Texas Red filter was used. Host genotype was determined based on DNA extracted from a fin clip devoid of red, transplanted cells.

### Creation of transgenic lines

The genomic and coding sequences of *msmo1* and *lss* were obtained from Wellcome Trust Sanger Ensembl database, *Danio rerio* (GRCz11). The open-reading frame (ORF) of *msmo1* was amplified from wild-type RNA; the ORF of *lss* was amplified from cDNA clone (NM_001083567.1) and cloned using Gateway BP reaction on the pDONOR221 plasmid (Invitrogen). The *fabp10* promoter was amplified from pHD157 (Addgene plasmid #84033) (Ni et al., 2012) using primers 5′-CTGAGCATCAGAATGGGGAAG-3′ and 5′-GCTCAACACAAAGTGAAGGTC-3′, and re-amplified with primers 5′-TGATATCGAATTCCTGCAGCCTGAGCATCAGAATGGGG-3′ and 5′-CGCTCTAGAACTAGTGGATCGCTCAACACAAAGTGAAG-3′ to direct proper orientation of the insert. Using NEBuilder HiFi DNA Assembly Master Mix, this product was then subcloned into the p5E-MCS (Addgene plasmid #26029) (Quillien et al., 2017) linearized with *Sma*I. Finally, plasmids were assembled using Gateway LR reaction and clones available in the Tol2kit (Kwan et al., 2007). To create the *hsp70l*:*msmo1*:IRESnlsEGFP plasmid, the ORF of *msmo1* was cloned between the *hsp70l* promoter and the IRESnlsEGFP sequence in the pDestTol2CG2 vector. To create the *fabp10:msmo1:pA* and *fabp10:lss:pA* lines, the ORF of *msmo1* or *lss* was cloned between the *fabp10* promoter and a polyA sequence in the pDestTol2CG2 vector. All transgenic lines were generated with the help of the Tol2 transposase method of transgenesis (Kawakami and Shima, 1999; Kwan et al., 2007). As the transgene backbone contains a *cmlc2*:EGFP element, all transgene-positive fish were identified at 2 dpf by EGFP-positive hearts.

### Heat-shock protocol

To induce transgene expression in *Tg(hsp70l*:*msmo1*:IRESnlsEGFP*)^nu99^* progeny of the identified *msmo1^nu81^* heterozygote crosses, fish were heat shocked daily at 42**°**C for 15 min, beginning at 24 hpf until collection and genotyping at 14 dpf.

### Histological sections

Two-month old fish were fixed overnight in phosphate-buffered 10% formalin and treated for 8 h before processing with 0.5 M EDTA, pH 8. Samples were processed using an STP 120 Spin Tissue Processor (Thermo Fisher Scientific). Dehydration steps were conducted gradually with 10%, 30%, 50%, and 70% ethanol, each for 1 h. Final ethanol treatments of 95% and 100% were each performed twice, both for 1 h each. Xylene was applied twice, 45 min each time, and paraffin (Histoplast Paraffin LP 8332; Thermo Fisher Scientific) applied three times for 60 min each time. Specimens were embedded using a Microm EC350-2 tissue-embedding center. All tissue samples were sectioned at 5 μm using a rotary microtome.

### *In situ* hybridization

The 918 bp template for the *msmo1* anti-sense RNA probe was amplified from wild-type RNA using a primer tailed with the T7 RNA polymerase promoter (5′-TGGTCTTATGTGAACTTTCTTTACA-3′ and 5′-TAATACGACTCACTATAGGGGTCTGTCCCACCAGGTGAAT-3′). Whole-mount *in situ* hybridization was performed as previously described (Thisse and Thisse, 2008), using low-stringency conditions. Hybridization mix was supplemented with 5% dextran sulfate to increase signal intensity (Lauter et al., 2011; Thisse and Thisse, 2014). RNAscope *in situ* hybridization was carried out on formalin-fixed, paraffin-embedded (FFPE) 5-μm sections. All RNA probes, hybridization kits and hybridization equipment, including ACD HybEZ Hybridization oven, were obtained from Advanced Cell Diagnostics (Newark, CA, USA). All FFPE sections were treated with an RNAscope 2.5 Universal Pretreatment Reagents kit and RNAscope 2.5 High Definition (HD)-Red Assay kit, as per the manufacturer’s recommendations, with the following adjustments to the tissue pre-treatment protocol. Slides were heated to 98-102**°**C in 1× Target Retrieval Reagents for 8 min and incubated at 40**°**C in Protease Plus for 15 min. Staining intensity for each probe was adjusted by varying the incubation time of Hybridization AMP 5. Signal detection time for each probe was as follows: 5 min for *col2a1a* (#409471) and *col10a1a* (#409481) or 30 min for *msmo1* (#438981), *ihha* (#490681), *ihhb* (#490691) and *ptch1* (#490701). The probe *dapB* (#310043) was included as a negative control in each assay, and incubated for either 5 min or 30 min depending on the assay. Each probe was tested in a minimum of three independent experiments on multiple sections. Slides were counterstained with 50% Hematoxylin and 0.02% (w/v) ammonia water, as per the manufacturer's suggestions. Photographs were taken using a Zeiss Axioplan 2 stereomicroscope equipped with a Zeiss Axiocam camera.

### Bone and cartilage staining

Larvae and adult fish were euthanized by submersion in ice-cold system water and fixed in phosphate-buffered 10% formalin for 2 h (larvae) or 1-2 days (adults). After rinsing in PBS, specimens were dehydrated gradually to 70% ethanol for long-term storage at 4**°**C. Whole-mount skeletal stains for adult bone and cartilage were performed as previously described (Taylor and Vandyke, 1985). Bone-only or cartilage-only protocols were performed for larval fish as previously described (Detrich et al., 2004), with the following modification: larvae were stained in a 0.005% solution of Alizarin Red dissolved in 1% KOH overnight. When not in use, specimens were kept in the dark to avoid fading. Photographs were taken using Zeiss Stemi 2000-CS and Zeiss Axioplan 2 stereomicroscopes, both equipped with a Zeiss Axiocam camera.

### Genotyping

For all genotyping protocols, small DNA fragments were amplified using the primers listed in Table S1. The *kol^nu7^* genotyping protocol was developed based on the polymorphism in linked exon 5 of *ndst3* that eliminates a *Sac*I restriction enzyme site. The genotyping protocols for *ndst3^nu20^*, *ugt8^nu82^* and *spock3^nu83^* were based on the elimination of restriction enzyme sites (Table 1). The genotyping of *msmo1^nu81^* and *lss^nu60^* alleles takes advantage of a 37 bp insertion and a 23 bp deletion, respectively. The PCR products were separated using either a 3.5% Metaphor agarose (Lonza) gel (*kol^nu7^*, *spock3^nu83^*, *msmo1^nu81^*, *lss^nu60^*) or 2.0% agarose gel (*ndst3^nu20^* and *ugt8^nu82^*).

### Statistical analysis

All statistical calculations were carried out with GraphPad Prism using the Chi-square or *t*-test calculator as noted.

### Zebrafish lines and maintenance

All fish used in this study were raised and cared for in accordance with an approved protocol by Ann and Robert H. Lurie Children's Hospital of Chicago Institutional Animal Care and Use Committee (IACUC; #13-036), and complied with National Institutes of Health standards provided in the ‘Guide for the Care and Use of Laboratory Animals’. The following wild-type, mutant and transgenic fish lines were used: AB (ZFIN ID: ZDB-GENO-960809-7), TU (ZFIN ID: ZDB-GENO-990623-3), *Tg(ubi:Zebrabow-M)^a131^* (ZFIN ID: ZDB-ALT-130816-2) (Pan et al., 2013), *kol^nu7^*, *msmo1^nu81^*, *lss^nu60^*, *ndst3^nu20^*, *ugt8^nu82^*, *spock3^nu83^*, *Tg(hsp70l*:*msmo1*:IRESnlsEGFP*)^nu99^*, *Tg(fabp10:msmo1:pA)^nu100^* and *Tg(fabp10:lss:pA)^nu101^*. All mutant fish described in the experiments are the results of crosses between heterozygous parents. The *kol^nu7^* mutant line is the result of an uncharacterized, naturally occurring mutation.

### Fish harvesting

Fish were collected at various developmental stages and SL was measured from snout to the end of the hypuralia, with any post-fixation adjustments applied to SL as necessary (Parichy et al., 2009).

## Supplementary Material

Supplementary information
