# Supplementary material for: Zebrafish models of skeletal dysplasia induced by cholesterol biosynthesis deficiency
Source: Dis Model Mech. 2020 Jun 24;13(6):dmm042549. doi: 10.1242/dmm.042549 (PMC7328163; doi:10.1242/dmm.042549)
Supplement: Supplementary information [file dmm-13-042549-s1.pdf]

Fig. S1

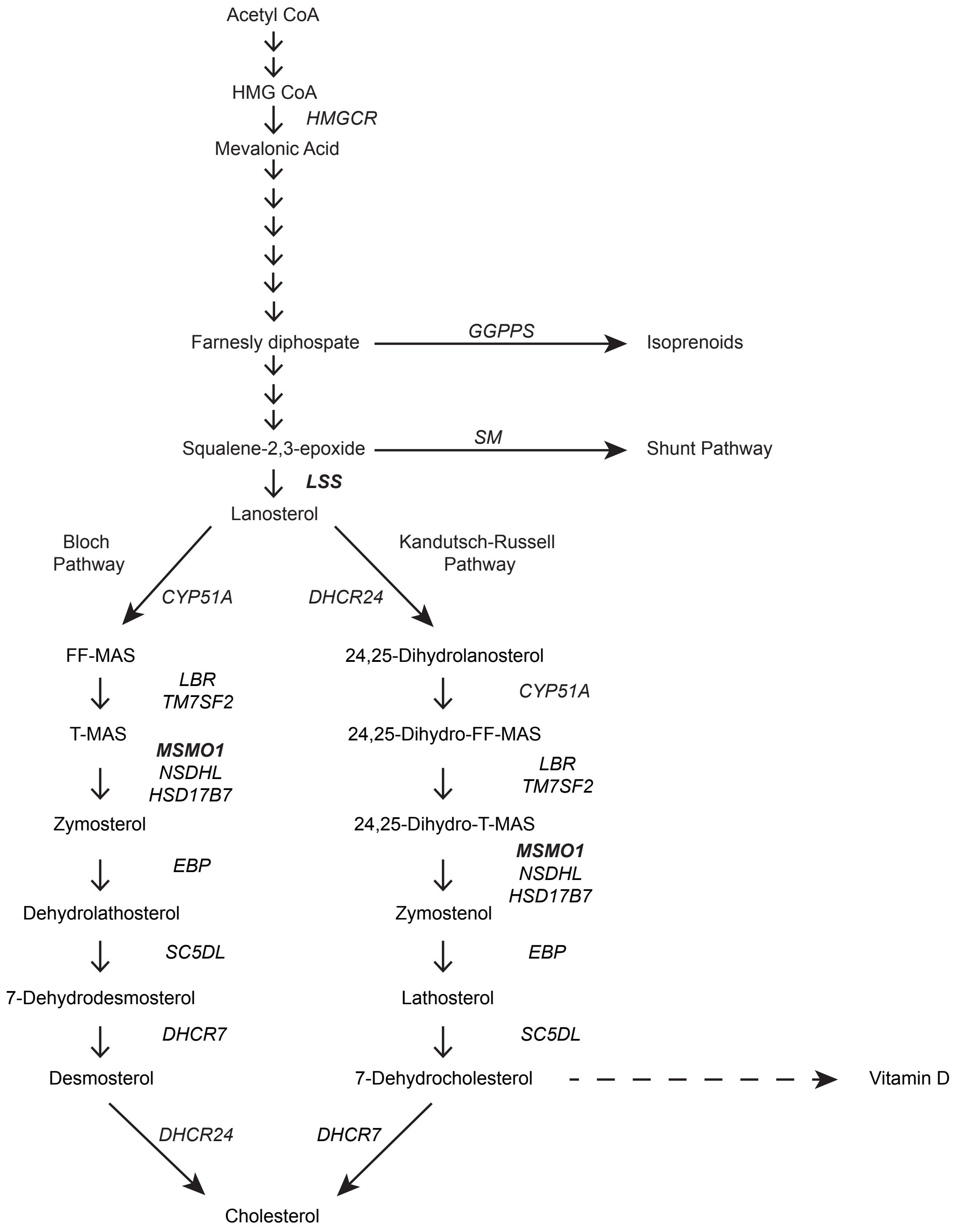

Disease Models & Mechanisms • Supplementary information

Fig. S1. Overview of the cholesterol biosynthesis pathway. Lanosterol synthase (LSS) is responsible for formation of the first sterol. Methylsterol monooxygenase 1 (MSMO1) catalyzes the removal of a methyl group from C4-methlysterols during the post-squalene cholesterol biosynthesis pathway.

Disease Models & Mechanisms • Supplementary information

Fig. S2

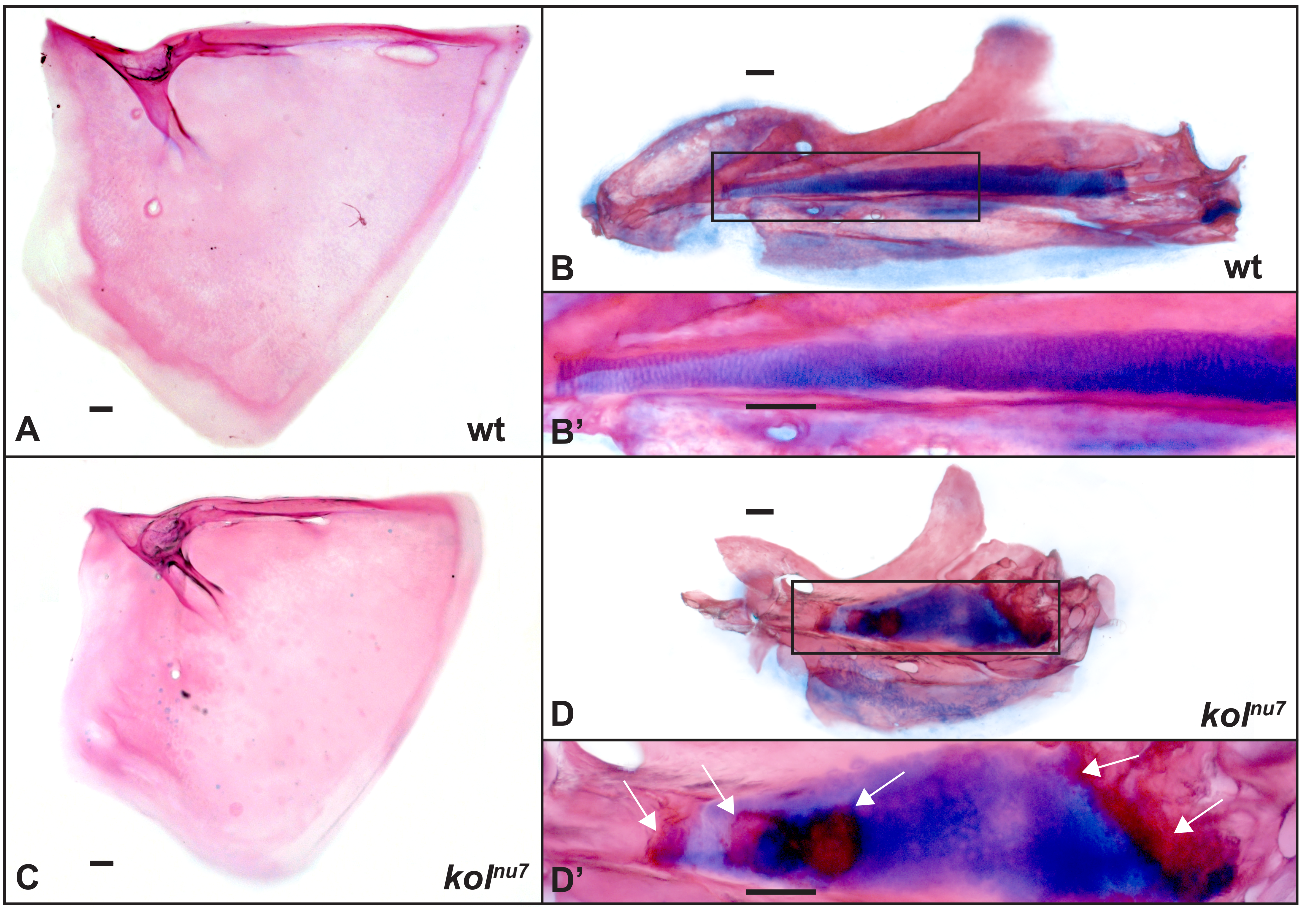

Fig. S2. Isolated intramembranous bones appear relatively unaffected in *koliber*^{nu7} while those located next to cartilage elements are deformed. The *kol*^{nu7} opercle bone (C) does not display ectopic ossification and appears similar in shape to that of wild type (A). Please note the *kol*^{nu7} opercle bone appears smaller in size due to adult *kol*^{nu7} mutants being smaller in size than wildtype siblings. The *kol*^{nu7} dentary bone is severely misshapen (D) with ectopic ossification within Meckel's cartilage, marked with arrows (D'). Scale bars=100µm. WT n=6; *kol*^{nu7} n=6.

Fig. S3

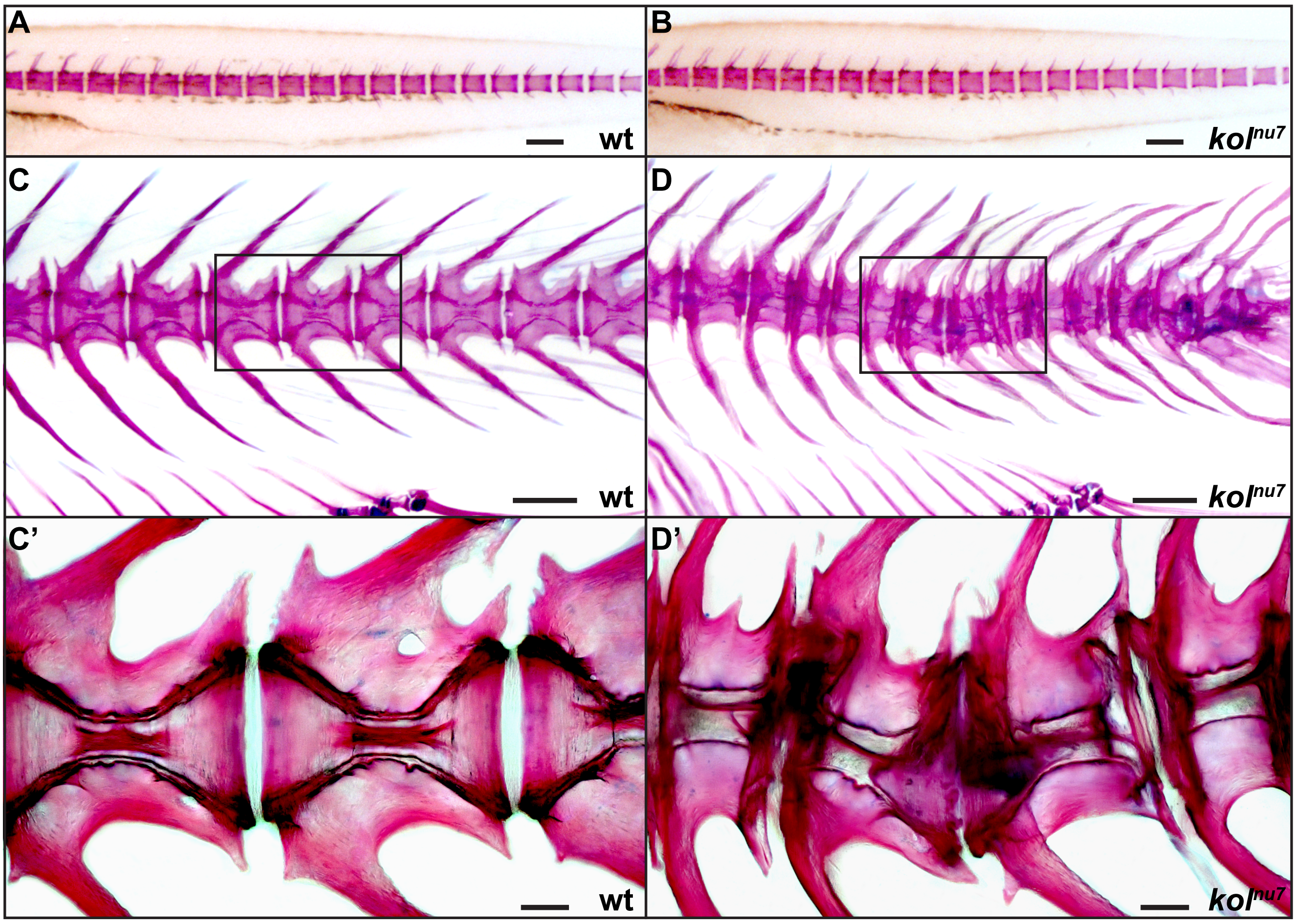

Fig. S3. Despite initial normal patterning of the ossification centra, adult *koliber^{nu7}* is characterized by numerous vertebral fusions At 6.3mm SL (~12dpf), centra ossification within *kol^{nu7}* is unaffected (B) and mirrors that of wild type (A). WT n=3; *kol^{nu7}* n=5. By four months of age, multiple vertebral fusions are present in *kol^{nu7}* (D, D'). WT n=4; *kol^{nu7}* n=5. Scale bars= 500μm (A,B,C,D); 100μm (C',D').

Disease Models & Mechanisms • Supplementary information

Fig. S4

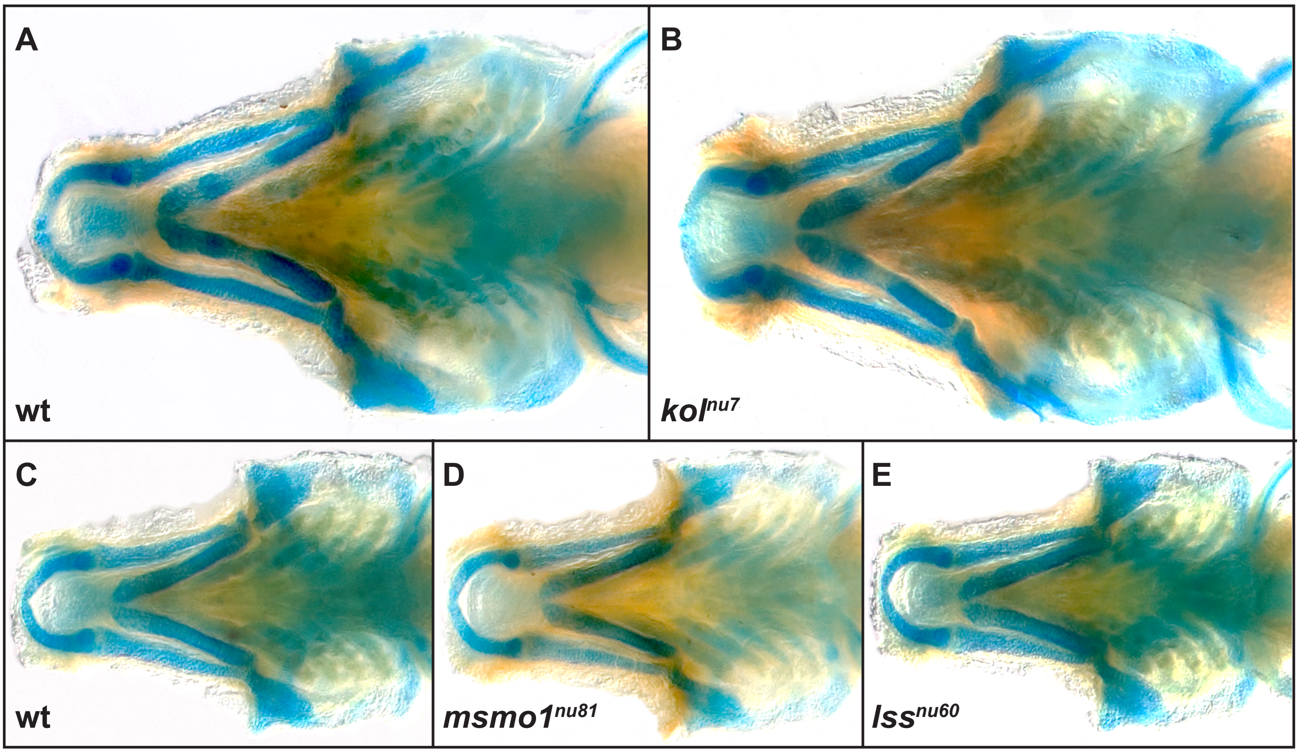

Fig. S4. Alcian blue staining of cartilage reveals initial patterning is unaffected in *koliber*^{nu7}, *msmo1*^{nu81}, and lss^{nu60}. Wild type (A) and *kol*^{nu7} (B) at ~4.7 mm SL (~8dpf). WT n=3; *kol*^{nu7}n=5. Wild type (C), *msmo1*^{nu81}(D), and lss^{nu60} (E) at ~3.5 mm SL (~5dpf). WT n=3 ; *msmo1*^{nu81} n= 3; lss^{nu60} n=4.

Disease Models & Mechanisms • Supplementary information

Fig. S5

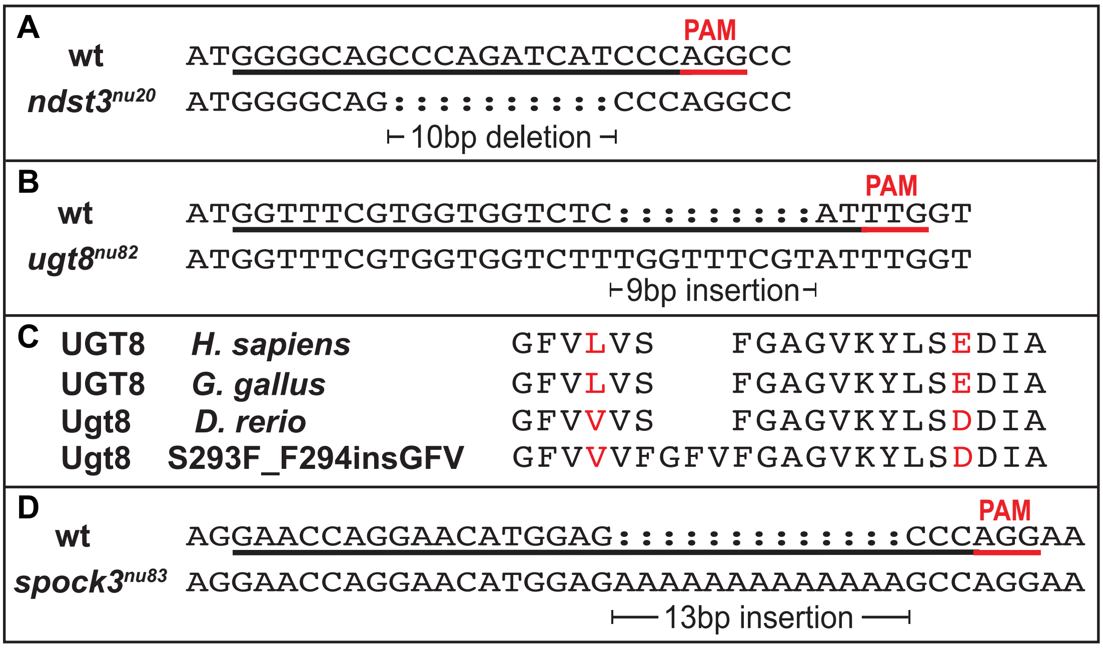

Fig. S5. Details of the isolated mutations in the genes within the *kol^{nu7}* critical region. (A) The *ndst3^{nu20}* allele is the result of a 10bp deletion. (B) The *ugt8^{nu82}* allele is the result of a 9bp insertion. (C) Multisequence alignment of evolutionarily highly conserved domain disrupted by *ugt8^{nu82}* mutation. The underlined serine corresponds to S293 of zebrafish Ugt8. Amino acids fully conserved between human (*H. sapiens*), chicken (*G. gallus*), and zebrafish (*D. rerio*) marked in black, substitution of base with similar character marked in red. (D) The *spock3^{nu83}* allele is the result of a 13bp insertion. Sequences targeted by gRNA underlined, PAM sequence marked in red.

Disease Models & Mechanisms • Supplementary information

Fig. S6

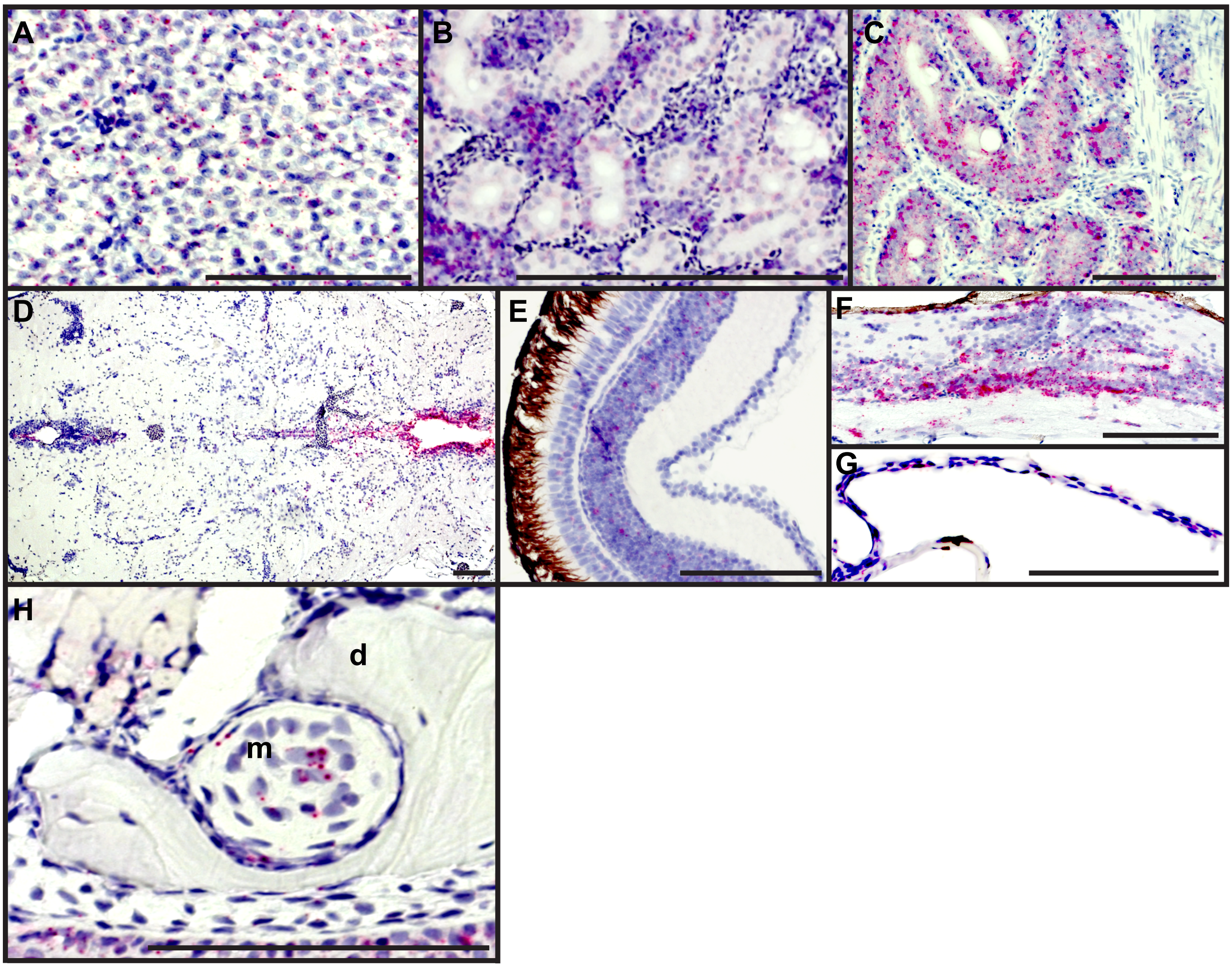

Fig. S6. Characterization of *msmo1* expression during juvenile development. Expression of *msmo1* is seen within the liver (A), kidney (B), intestine (C), brain (D), retina (E), spinal cord (F), skin (G), and Meckel's cartilage (m) (H). Expression of *msmo1* is undetectable in the intramembranous dentary bone (d) (H). Images represent *msmo1* expression analyzed using RNAscope *in situ* hybridization on paraffin sections of juvenile wildtype fish at approximately two months of age (SL ~15mm). Scale bars=100μm. WT n=3.

Table S1 Genotyping Primers

Allele	F Primer (5'-3')	R Primer (5'-3')
kol^{nu7}	CCGTCAGACATGGGGTTTAT	CTCACAGGATTGAGCAGGAC
ndst3^{nu20}	TATTCTCAGCTTGGACAGGACA	AGCTTTCTGCCTGTTAAAAACG
ugt8^{nu82}	TGTTGCATTTCAGCCATTTT	AATGGACTGAGACGCAGGTG
spock3^{nu83}	AAGTTCAGTTAATCTAACTCGTCTGTT	TTTCCAACTTTAAACAAAGTGATGTC
msmo1^{nu81}	TCATTCAAGCCTTTCTGTGC	TTTGCGGAAGAAATGAATCC
lss^{nu60}	TGAGTAATGTGGACGGCAGA	TGCCTTGTGTATCATCAGA

Disease Models & Mechanisms • Supplementary information
